# Vibration signal analysis for rolling bearings faults diagnosis based on deep-shallow features fusion

**DOI:** 10.1038/s41598-025-93133-y

**Published:** 2025-03-18

**Authors:** Ahmed Chennana, Ahmed Chaouki Megherbi, Noureddine Bessous, Salim Sbaa, Ali Teta, El Ouanas Belabbaci, Abdelaziz Rabehi, Mawloud Guermoui, Takele Ferede Agajie

**Affiliations:** 1 Dept. Electrical Engineering, Laboratory of LI3C, University of Mohamed Khider, Biskra, Algeria; 2 Dept. Electrical Engineering, Laboratory of LGEERE, University of El Oued, El Oued, Algeria; 3 Dept. Electrical Engineering, Laboratory of VSC, University of Mohamed Khider, Biskra, Algeria; 4 Dept. Electrical Engineering, Laboratory of LAADI, University of Djelfa, Djelfa, Algeria; 5 Laboratory of Medical Informatics (LIMED), Faculty of Technology, University of Bejaia, 06000 Bejaia, Algeria; 6 Telecommunications and Smart Systems Laboratory, University of Djelfa, PO Box 3117, 17000 Djelfa, Algeria; 7 Unite de Recherche Appliquée en Energies Renouvelables, URAER, Centre de Développement des Energies Renouvelables, CDER, 47133 Ghardaïa, Algeria; 8 Department of Electrical and Computer Engineering, Faculty of Technology, Debre Markos University, P. BOX 269 Debre Markos, Ethiopia

**Keywords:** Bearing fault diagnosis, Vibration signals, Transfer learning, Shallow descriptor, Deep features, MBH-LPQ, VGGish, CNN, Energy science and technology, Engineering, Mathematics and computing, Physics

## Abstract

In engineering applications, the bearing faults diagnosis is essential for maintaining reliability and extending the lifespan of rotating machinery, thereby preventing unexpected industrial production downtime. Prompt fault diagnosis using vibration signals is vital to ensure seamless operation of industrial system avert catastrophic breakdowns, reduce maintenance costs, and ensure continuous productivity. As industries evolve and machines operate under diverse conditions, traditional fault detection methods often fall short. In spite of significant research in recent years, there remains a pressing need for improve existing methods of fault diagnosis. To fill this research gap, this research work aims to propose an efficient and robust system for diagnosing bearing faults, using deep and Shallow features. Through the evaluated experiments, our proposed model Multi-Block Histograms of Local Phase Quantization (MBH-LPQ) showed excellent performance in classification accuracy, and the audio-trained VGGish model showed the best performance in all tasks. Contributions of this work include: Combine the proposed Shallow descriptor, derived from a novel hand-crafted discriminative features MBH-LPQ, with deep features obtained from VGGish pre-trained of Convolutional Neural Network (CNN) using audio spectrograms, by merging at the score level using Weighted Sum (WS). This approach is designed to take advantage of the complementary strengths of both feature models, thus enhancing overall bearing fault diagnostic performance. Furthermore, experiments conducted to verify the approach’s performance is assessed based on fault classification accuracy demonstrated a significant accuracy rate on two different noisy datasets, with an accuracy rate of 98.95% and 100% being reached on the CWRU and PU datasets benchmark, respectively.

## Introduction

Efficient early fault diagnosis in rotating machinery is crucial for reducing maintenance expenses, ensuring equipment availability and reliability, and expediting decision-making regarding replacements and repairs, thereby enhancing productivity and safety^1^. Maintenance strategies are typically classified into three main categories: corrective maintenance (CreM), preventive maintenance (PveM), and predictive maintenance (PdiM). CreM is very costly because it is only implemented after machines break down, requiring production to stop for replacing defective parts. PveM is the process of performing regularly planned and scheduled maintenance tasks to prevent unexpected breakdowns in the future. Although effective, it often requires routine preventative measures that may not be unnecessary, resulting in increased maintenance costs. PdiM relies on continuous monitoring of equipment condition through the use of predictive Artificial Intelligence (AI) techniques to track and evaluate the functional integrity of equipment operations in real time. These approaches enable maintenance actions to be predicted based on the actual condition of the equipment. Pdim is one of the most important maintenance strategies, as it helps extend the operational life of the machines and maintain their performance levels^2^.

**Fig. 1:**
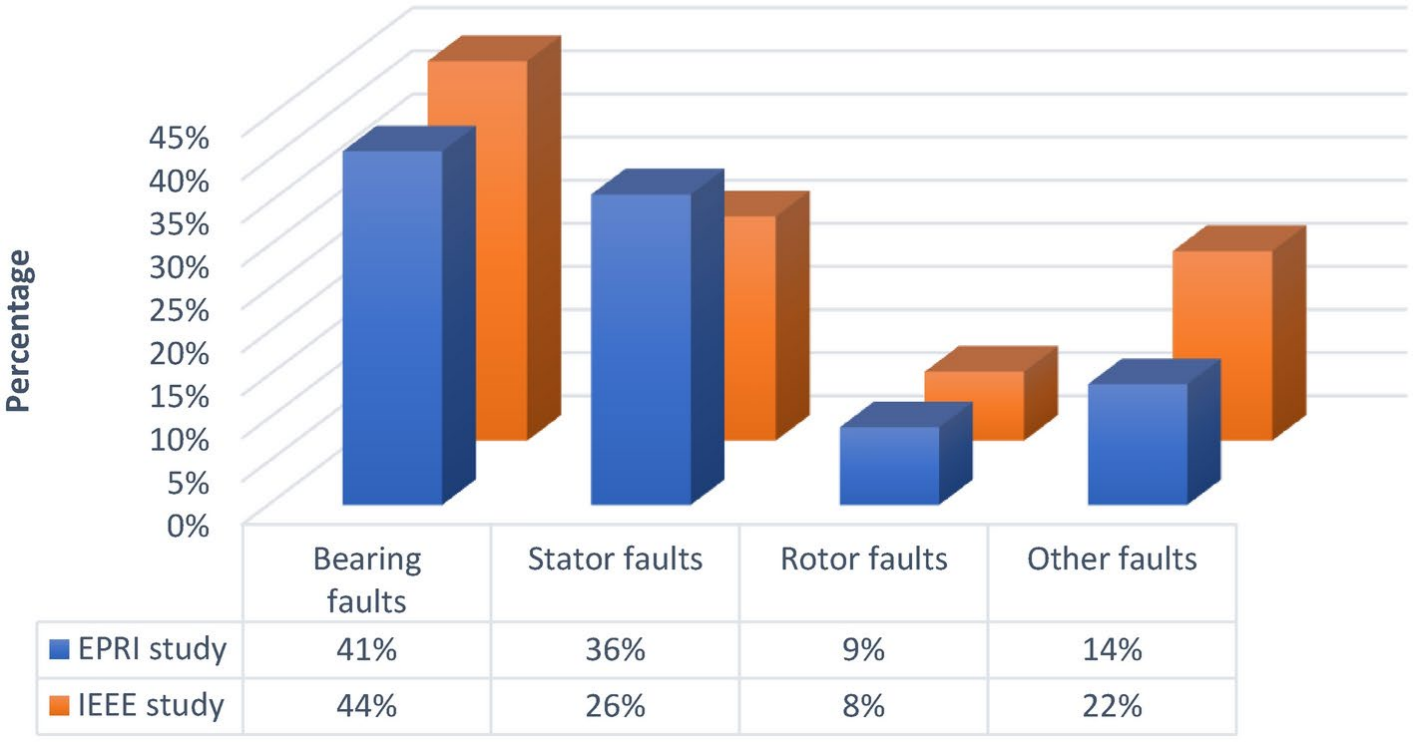
IEEE and EPRI faults studies.

Fault diagnosis systems mainly focus on detecting, isolating and identifying various faults, and with the advancement of technology, the ability to store system operating data has evolved, leading to the enhancement of traditional diagnostic methods and the emergence of artificial intelligence techniques. These AI techniques enable the analysis of system data, resulting in more effective and accurate fault diagnosis^3^. Failures of rotating machines, including induction motors, can be categorized into four main categories: Rotor faults, bearing faults, stator-related faults, and other faults^4^. The bearing is a crucial component in rotating machinery, playing a vital role in its operation. According to induction motor reliability studies by IEEE and Electric Power Research Institute (EPRI), bearing faults are listed as the leading cause of failure, accounting for 41% to 44% of reported faults. Figure 1 shows the different percentages of faults according to the EPRI and IEEE standards^5–9^.

Rolling element bearings (REBs) are often used under extremely harsh conditions, including high pressures, high speeds, and high temperatures, which expose them to various failure states that can lead to system downtime, significant economic losses, and serious accidents^10–12^. Bearing deterioration can lead to vibrations that degrade the machine’s performance and affect its ability to bear loads and perform tasks effectively, thus reducing the life of the machine. Without promptly maintenance, these issues can escalate into catastrophic malfunctions. Such malfunctions not only result in extended machine downtime and high repair costs but also pose significant safety risks in the work environment^13,14^.

Condition monitoring using vibration analysis approach is attractive due to its non-intrusive sensors, relatively low expense, and ability to detect wide range of machine faults based on specific vibration patterns. The frequencies of these vibrations vary depending on the type and severity of the faults, while the intensity of these vibrations helps assess the severity of the problem. Such information enables the implementation of appropriate maintenance procedures based on the vibration analysis of the machines^15^. A comprehensive and accurate evaluation of the severity of faults is crucial to achieving reliable and accurate diagnostic results^16,17^. Accurately extracting fault features is crucial for achieving accurate diagnosis and obtaining a comprehensive understanding of the nature and underlying causes of faults. This helps reducing maintenance time, enhance overall system performance, and lower long-term maintenance and operational costs^18^.

To prevent the negative effects caused by bearing degradation, it is crucial to monitor their health status and accurately forecast their remaining useful lifetime (RUL). This proactive approach facilitates the timely execution of predictive maintenance strategies aimed at restoring bearings to optimal performance levels^19,20^. With an increasing focus on improving the reliability of industrial products, the Prognostics and Health Management (PHM) approach has emerged as a pivotal technology in the Fourth Industrial Revolution. It enhances the operational safety and cost-effectiveness of engineering systems by preventing sudden failures and potential risks. This approach is integral for ensuring the availability and reliability of industrial products and systems, encompassing fault detection, identification of fault type and location (fault diagnosis), and prediction of future health status and remaining useful lifetime of machines.By addressing these areas, PHM extends system longevity and performance, reduces unplanned downtime, and lowers maintenance costs, establishing itself as a cornerstone of modern industrial maintenance practices^21–23^.

In the modern industrial era, there is an urgent need for intelligent systems capable of providing accurate and efficient fault diagnosis. These systems leverage advanced technologies such as machine learning, deep learning, and pattern recognition to significantly improve fault detection accuracy, facilitating the implementation of predictive maintenance, and enhancing the ability to efficiently diagnose faults. Such technological advancements are especially crucial for the early detection of potential problems in roller bearings, which are essential components in many mechanical systems. Early detection of bearing failures helps prevent sudden breakdowns, reducing downtime, and optimizing maintenance schedules. Therefore, a robust and effective fault diagnosis system is indispensable to support proactive maintenance strategies, leading to increased productivity, reduced maintenance costs and improved overall system performance. The integration of these smart technologies not only ensures the reliability and longevity of industrial machines, but also enhances production efficiency, reduces costs, and contributes to improving the overall performance of industrial systems^24^.

In recent years, technological advancements have led to a significant leap in relying on shallow and deep learning-based diagnosis methods across wide range of fields, with particular emphasise on machinery faults diagnosis^23^ ,^25,26^. Among these advancements,the diagnosis of faults in induction motors has remarkably benefited from deep learning techniques. These methods are distinguished not only by their ability to discover hidden patterns in complex data, but also by their ability to provide accurate diagnoses even in cases where signs of malfunction are invisible. This progress has increased the confidence of both academia and industry regarding the adoption of deep learning techniques to develop advanced diagnostic systems that are highly accurate and reliable. The ability to handle and analyze large amounts of data using advanced methods enables these systems to predict malfunctions before they occur. This capability helps improve maintenance efficiency, reduce unplanned downtime, and positively influences overall productivity.

Effective integration of shallow and deep learning methods is necessary to leverage the strength of both shallow and deep feature extraction methods in order to obtain an effective and robust diagnostic model. This article proposes a novel method for diagnosing bearing faults in motors using vibration signals. The proposed model incorporates feature learning based on both shallow features (Sh-F) and deep features (D-F). Processed vibration signals are converted into visual representations, which are then used as inputs for features extraction and classification using both shallow and deep feature extraction models. To avoid missing features and enhance classification accuracy, we introduce a new shallow descriptor called Multi-Block Histograms of Local Phase Quantization (MBH-LPQ). This descriptor is designed to be integrated with the most powerful classifier in feature-based deep classification models. Additionally, spectrograms, which provide a time-frequency analysis of vibration signals, are widely utilized in deep learning frameworks. However, the application of visual texture features technique within shallow learning models, which rely on manually extracted features, has not been thoroughly investigated. To the best of our knowledge, researches on applying visual texture feature techniques (Shallow features) to spectrum images for bearing faults diagnosis is still rare. Our research addresses this gap by introducing an innovative method for bearing fault diagnosis that significantly enhances both accuracy and efficiency. This method incorporates a sequence of sophisticated pre-processing techniques, converting vibration signals into visual representations, and extracts distinctive features from spectrogram images. It also leverages cutting-edge deep learning algorithms and visual texture feature techniques.

We have made several key contributions and conducted extensive testing to validate the proposed bearing faults diagnosis method using two challenging open-source datasets provided by: Case Western Reserve University (CWRU), USA^27^ and University of Paderborn (PU), Germany^28^. This paper investigates a novel approach for bearing faults diagnosis that integrates shallow features and deep features extraction and fusing them as well. The proposed models demonstrates a superior capability for bearing faults diagnosis. The principal contributions of our study are summarized as follows:

1. We introduce a new shallow feature descriptor named Multi-Block Histograms of Local Phase Quantization (MBH-LPQ). This descriptor captures the structural details of spectrogram images by segmenting them into *b* sub-blocks.
2. The proposed diagnosis method utilizes a non-linear subspace learning approach, Exponential Discriminant Analysis (EDA). This approach is carefully designed to address the complexities of bearing fault diagnosis.
3. We implement a score-level fusion through Weighted Sum (WS) to improve the accuracy of the proposed diagnosis model. This fusion strategy combines shallow features derived from the proposed MBH-LPQ descriptor with deep features obtained from pretrained VGGish. By leveraging the complementary strengths of both feature types, this approach aims to improve overall performance.

The remainder of this paper is structured as follows: the second section 2 reviews the related work in the field. The third section 3 details the methodology employed in this study, including a description of the proposed MBH-LPQ descriptor. The fourth section 4 outlines the experimental setup and provides a comprehensive analysis and discussion of the results. Finally, The last section 5 concludes the paper and suggests directions for future research.

## Related works

In recent years, deep learning has become an effective means of addressing various diagnostic challenges, especially in the field of image processing. However, traditional computer vision techniques, which have been steadily evolving by researchers, continue to receive attention from researchers^29^. Where the use of traditional techniques derived mainly from hand-made features such as local binary pattern (LBP) technique to diagnose bearing faults as stated in the following studies:^30–34^. Many studies based on signal processing techniques have also been conducted, including time series analysis, frequency analysis, and time-frequency analysis using decomposition, deconvolution, and wavelet transform methods^35–41^. However, these techniques still face the challenges such as noise suppression, which leads to loss of fault features information. Therefore, to address the various challenges facing traditional diagnostic techniques, the trend towards using deep learning was a top priority, as many algorithms have been developed to diagnose bearing faults using deep learning. Although these techniques have led to a qualitative leap in the world of fault diagnosis in the era of technology, there is still a need for traditional techniques, which forced researchers to combine the two approaches, as stated in Wang’s study^42^, where to improve the accuracy of diagnosing bearing faults, the author presented a method that combines the Slime Mould Algorithm (SMA), Variational Mode Decomposition (VMD), and a Convolutional Neural Network-Long Short-Term Memory (CNN-LSTM) model. This integrated approach has shown significant superiority in prediction accuracy over using each model independently. Chang et al.^43^, introduced a novel approch based on Osprey-Cauchy-Sparrow Search Algorithm (OCSSA) and improved variational mode decomposition combined with convolutional neural network bidirectional long short-term memory (CNN-BiLSTM). This method called OCSSA-VMD-CNN-BiLSTM. This research tackles the difficulties presented by high levels of noise and the nonstationary nature of vibration signals, aiming to improve the effectiveness and accurate identification of fault diagnosis for rolling bearings in electric motors. Nishat and Kim^44^, presented a model framework for bearing fault classification based on motor current signal where features were extracted using Discrete Wavelet Transform (DWT) and then the bearing fault condition was classified using two ensemble Machine Learning (ML) classifiers extreme Gradient Boosting (XGBoost) and Random Forest (RF). The authors demonstrated that this method is effective in fault classification. Siddique et al.^45^, presented a novel methodology for diagnosing bearing faults using Mel-transformed scalograms derived from vibration signals. The process involves dividing the signals into windows and processing them through a Mel filter bank to convert them into a Mel spectrum, followed by accurate classification using an artificial neural network optimized with the FOX optimizer algorithm. Naimat et al^46^. ,investigated the use of CWT to convert raw data into two-dimensional images for diagnosing three types of faults: bearing, tool, and gear faults. In their study, a modified AlexNet architecture was employed for feature extraction, while the ant colony optimizer (ACO) was utilized to retain the most discriminative features.

## Proposed methodology

**Fig. 2:**
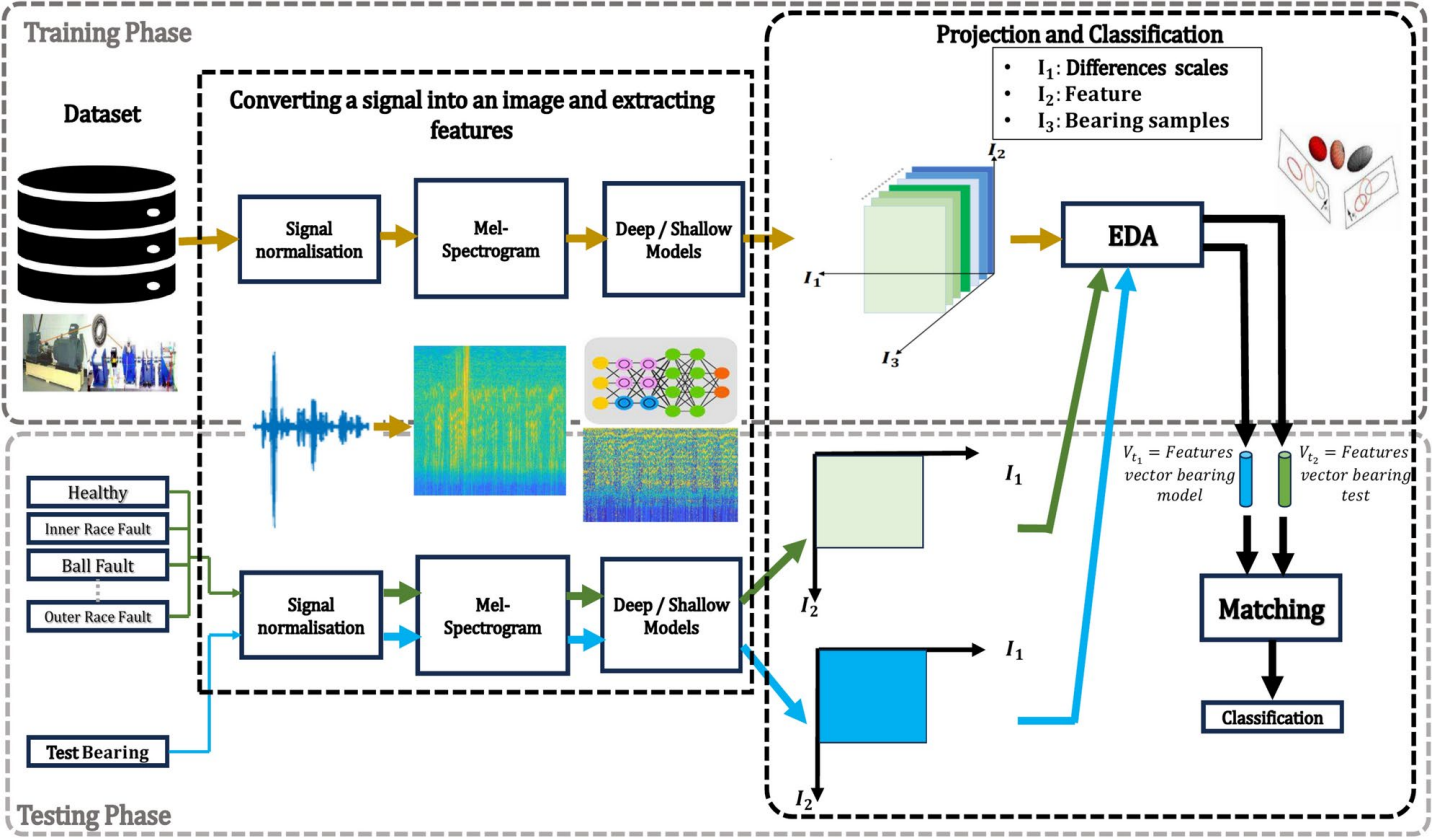
Block diagram of the proposed fault diagnosis method.

**Fig. 3:**
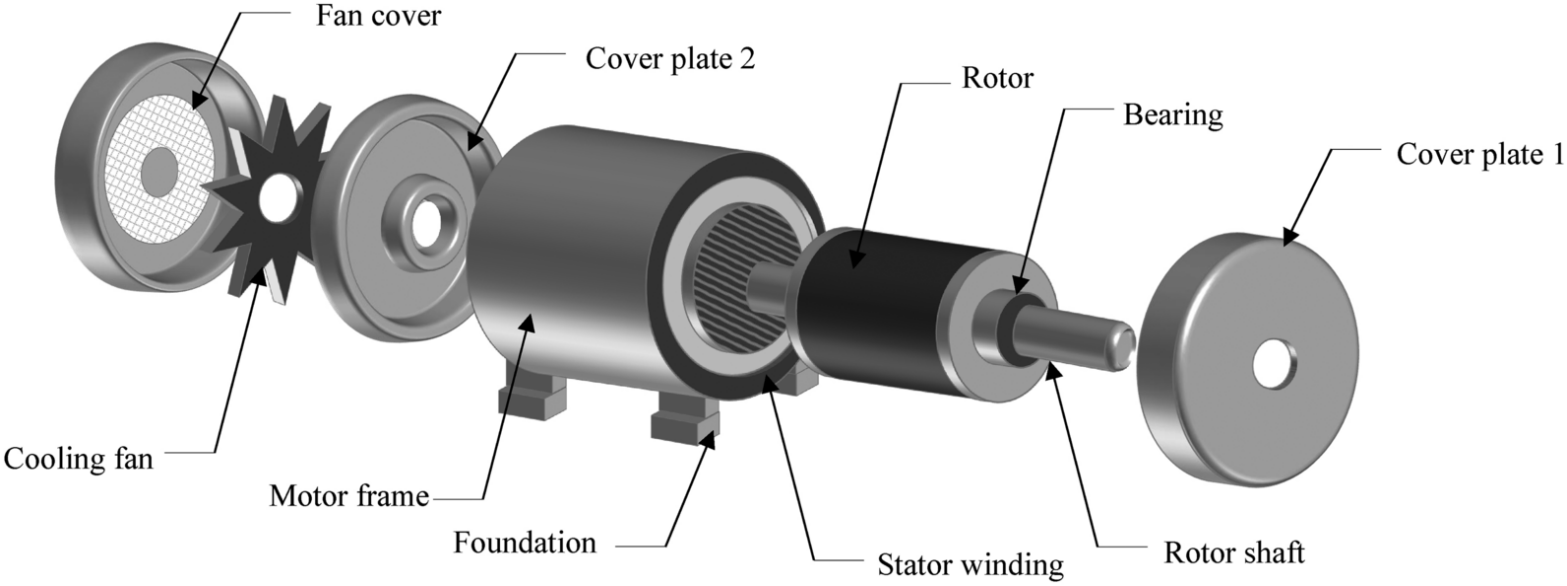
Structure of an induction motor.

Our bearing fault diagnosis framework, illustrated in Figure 2, comprises five essential stages. This section provides a detailed explanation of each stage. The process begins with data pre-processing of healthy and faulty signals collected from induction motor parts depicted in Figure 3, where continuous vibrational waveforms are normalized and segmented into samples, which are then divided into training (80%) and testing (20%) sets. These signals are subsequently converted into visual data representations using Log Mel-spectrograms. For feature extraction, we employ a combination of shallow texture descriptors, including the newly proposed MBH-LPQ descriptor, alongside pre-trained deep CNN models such as VGG16 (trained on images) and VGGish and YAMNet (trained on audio). After feature extraction, we utilize Exponential Discriminant Analysis (EDA) for dimensionality reduction and classification, with cosine similarity used for comparison. In the final stage, we combine the MBH-LPQ descriptor that achieved the highest classification accuract among the various shallow texture descriptors with the best-performing CNN model (VGGish), through a Weighted Sum (WS) at the score level, enhancing the overall diagnostic accuracy. This section offers an in-depth explanation of each stage.

### Log Mel-Spectrogram representation of continuous vibrational waveforms

Continuous vibrational waveforms are converted into spectrograms using Short-Time Fourier transform (STFT) and log transformation to obtain a slope spectrogram. A spectrogram represents the frequency content over time of a given signal. A slope spectrogram converts a logarithmic scale to high frequencies while maintaining a linear scale to low frequencies. Spectral representation reduces frequency components to a specified number of slope frequencies. After initial tests, the spectrogram representation was preferred due to its compact frequency dimension, with most errors located within the low frequency range^47^. In this subsection, the crucial stages of data pre-processing are outlined. The foremost step prior to the transformation of signal vibration data into an image format is normalization. Inherent variations in the amplitude of signal vibrations, even within the same signal category, arise from differing recording conditions. To achieve uniformity, we normalized the signal vibrations by subtracting the mean and then scaling the amplitude to fit within the range of [-1, 1], as detailed in Equation 1.

1 $$\begin{aligned} \mathrm {x=\frac{x-mean(x)}{max(abs(x))}} \end{aligned}$$

After normalization, the audio signal undergoes transformation into visual representations via the Mel-Spectrogram technique. The spectrogram X(k,t) is generated by applying the windowed STFT to the input signal, a process meticulously defined in Equation 2^48^.

2 $$\begin{aligned} X(m, t) = \sum _{n=0}^{N-1} x[n]w[n - t]e^{-\frac{2\pi imn}{N}}, \quad m = 0, \ldots , N-1 \end{aligned}$$

Within this context, *x*[*n*] denotes the input vibration signal, *N* signifies the length of the window, *w*[*n*] corresponds to the Hamming window function, and *m* represents the frequency index, which is measured in hertz (Hz).

The Mel spectrum is derived by applying the STFT to each frame, which converts the energy/amplitude spectrum from a linear frequency scale to a logarithmic Mel scale. The transformed data is then processed through a filter bank to extract the eigenvectors. For each tone with an actual frequency *f*, measured in Hertz (Hz), a perceived pitch is assessed on a scale termed the Mel scale, as specified in Equation 21.

3 $$\begin{aligned} f_{\text {mel}} = 2595 \cdot \log _{10}\left( 1 + \frac{f_{Hz}}{700\text {Hz}}\right) \end{aligned}$$

### Extraction of discriminant features

The extraction of discriminative visual features from images is a critical aspect of various recognition tasks, serving as the foundation for recognition systems and computer vision applications, particularly in the domain of vibration signal analysis^49^. In image classification, the quality of an image’s representation whether through local textural details or learned features plays a significant role in the effectiveness of the method^50^. Recent studies have demonstrated that visual features can outperform traditional audio features, such as Mel-Frequency Cepstral Coefficients (MFCC)^51^, Perceptual Linear Prediction (PLP)^52^, and Constant Q Cepstral Coefficients (CQCC)^53^, in signal-based classification tasks. In this context, we introduce a novel approach for extracting efficient and discriminative features from spectrogram images. Our method employs a fusion of deep and shallow features, specifically designed for bearing fault diagnosis. This is achieved using a Convolutional Neural Network (CNN) enhanced by transfer learning, combined with traditional shallow texture descriptors, including the proposed MBH-LPQ. A detailed description of each descriptor is provided below:

#### Traditional shallow texture descriptors

In this subsection, we elaborate on Three local descriptor methods of shallow features: Local Binary Patterns (LBP), Local Derivative Pattern (LDP) and Local Phase Quantization (LPQ).

**Fig. 4:**
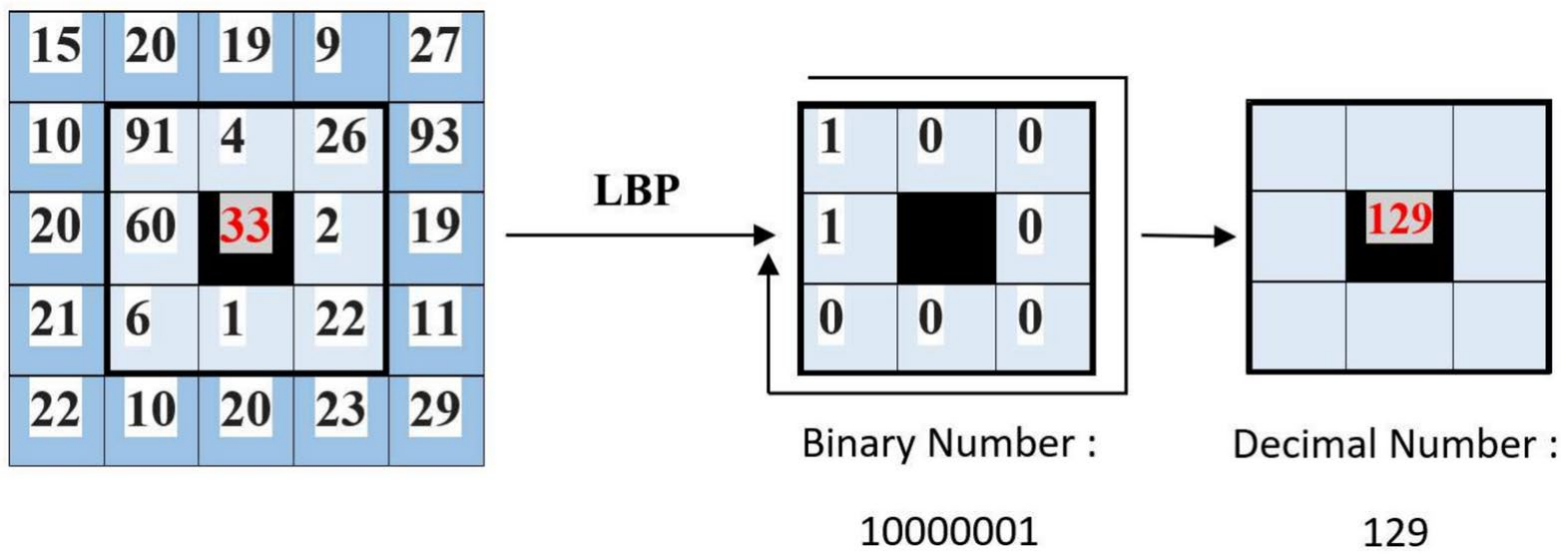
LBP Operator illustration.

**1. Local binary pattern (LBP)**^54^: LBP integrates the analysis of local structures with the analysis of occurrences. This method characterizes each image pixel, denoted as $q_c$, using a binary pattern. This pattern is derived from the difference in grey level values between the pixel $q_c$ and its surrounding pixels in a circular neighborhood, defined by a specified radius R centered at $q_c$ as illustrated in Figure 4. In the fundamental form of this approach, the LBP operator for a given neighborhood in the image is defined as follows:

4 $$\begin{aligned} LBP_{P,R}(q_c) = \sum _{p=0}^{P-1} s(x)2^p \end{aligned}$$

Where, *x* represents the difference between the intensity levels of the neighboring pixels, denoted as $q_p$ and the central pixel $q_c$, within a circular neighborhood characterized by a radius *R* and *P* neighboring pixels. Furthermore, *s*(*x*) is defined as:

5 $$\begin{aligned} s(x) = \left\{ \begin{array}{ccc} 1 & \text {if } x \ge 0 \\ 0 & \text {otherwise} \end{array} \right. \end{aligned}$$

**2. Local Derivative Pattern (LDP)**^55^: LDP is an extension of the Local Binary Pattern (LBP) that captures more complex local textures by considering directional information in the image. This descriptor encodes the relationship between a central pixel and its surrounding neighbors using directional derivatives, enhancing the ability to detect detailed patterns and making it more robust against noise.

**Fig. 5:**
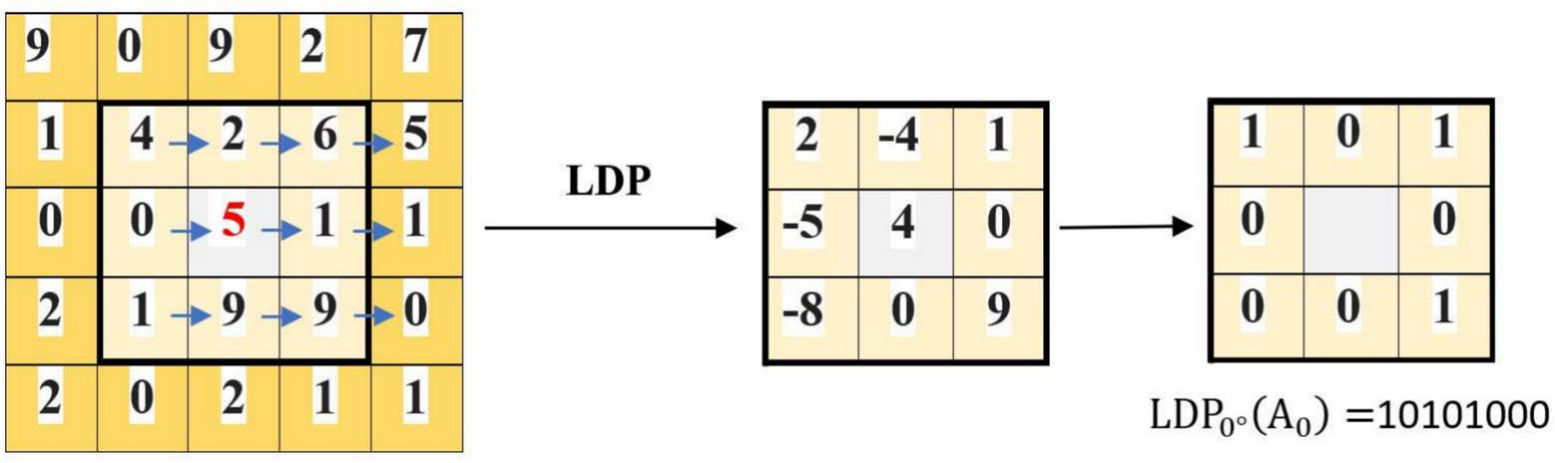
LDP Operator illustration.

The LDP operator works by calculating first-order derivatives of pixel intensities along specific directions ($0^{\circ }$, $45^{\circ }$, $90^{\circ }$, and $135^{\circ }$) relative to the central pixel $A_0$ as shown in Figure 5. The first-order derivatives are defined as follows:

6 $$\begin{aligned} I'_{\alpha }(A_0) = I(A_0) - I(A_i) \end{aligned}$$

where $I(A_0)$ and $I(A_i)$ are the intensity values of the central and neighboring pixels, respectively, and $\alpha$ represents the directional angle ($0^{\circ }$, $45^{\circ }$, $90^{\circ }$, $135^{\circ }$).

The second-order LDP, $LDP_{\alpha }^{2}(A_0)$, encodes the co-occurrence of these derivatives between the central pixel and each of its eight neighbors using a binary coding function. This function produces binary codes that reflect the gradient change patterns in a local region, providing a richer representation than LBP. Mathematically, the second-order LDP descriptor is given by:

7 $$\begin{aligned} LDP_{\alpha }^{2}(A_0) = \sum _{i=1}^{8} f(I'_\alpha (A_0), I'_\alpha (A_i)) \end{aligned}$$

where the function $f$ is defined as:

8 $$\begin{aligned} f(I'_\alpha (A_0), I'_\alpha (A_i)) = {\left\{ \begin{array}{ll} 0, & \text {if } I'_\alpha (A_i)> 0 \text { and } I'_\alpha (A_0) > 0 \\ 1, & \text {otherwise} \end{array}\right. } \end{aligned}$$

This binary pattern captures the directional intensity variations in the neighborhood, allowing LDP to effectively describe complex textures and improve performance in tasks like image recognition, where subtle variations are crucial.

**Fig. 6:**
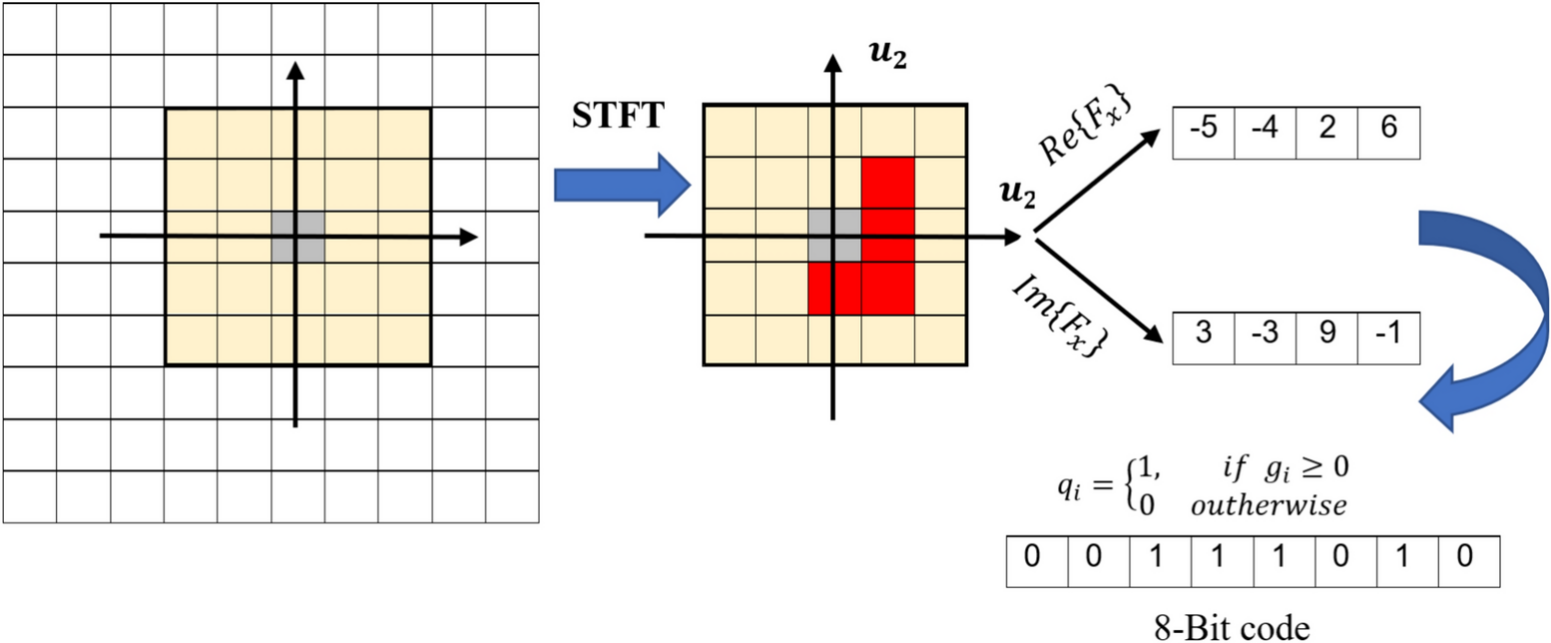
LPQ Operator illustration.

**3. Local Phase Quantization (LPQ)**^56^: The LPQ descriptor, is utilized as a spatial blur-insensitive texture analysis method. In the LPQ approach, the STFT is first applied to a blurred image, represented as $g(x, y)$. This transformation is performed over a local neighborhood defined by $N_x$ and $N_y$ with a size of $M \times M$ as shown in Figure 6. The transformation is given by the following equation:

9 $$\begin{aligned} F(u, v) = \sum _{x \in N_x} \sum _{y \in N_y} g(x, y)e^{-j2\pi \frac{(ux+vy)}{M}} \end{aligned}$$

In the LPQ method, four complex coefficients are extracted for each pixel, corresponding to specific frequency points: $u_1=(a,0)$, $u_2=(0,a)$, $u_3=(a,a)$, and $u_4=(-a,a)$. These coefficients are used to quantize the sign of the real ($\mathbb {R}$) and imaginary ($\mathbb {I}$) parts of each Fourier coefficient, as shown:

10 $$\begin{aligned} C= & [F(u_1), F(u_2), F(u_3), F(u_4)] \end{aligned}$$

11 $$\begin{aligned} K= & [\mathbb {R}\{C\}, \mathbb {I}\{C\}] \end{aligned}$$

The quantization is performed according to the rule:

12 $$\begin{aligned} q_i = {\left\{ \begin{array}{ll} 1, & \text {if } K_i \ge 0 \\ 0, & \text {if } K_i < 0 \end{array}\right. } \end{aligned}$$

where $a$ is a frequency parameter set to the smallest non-zero frequency, $1/M$. The resulting eight binary values, $q_i$, are then combined into a single integer in the range of 0 to 255 using the following equation:

13 $$\begin{aligned} f_{lpq} = \sum _{i=1}^{8} q_i 2^{i-1} \end{aligned}$$

This representation effectively encodes the local phase information of the image, making LPQ highly robust to image blurring while preserving important texture details.

#### Deep features based transfer learning

In this part, we elaborate on two types of deep features: YamNet and VGGish, both pre-trained using spectrogram images. Additionally, we provide details on the VGG16 model, which has been pre-trained on object images.

**Fig. 7:**
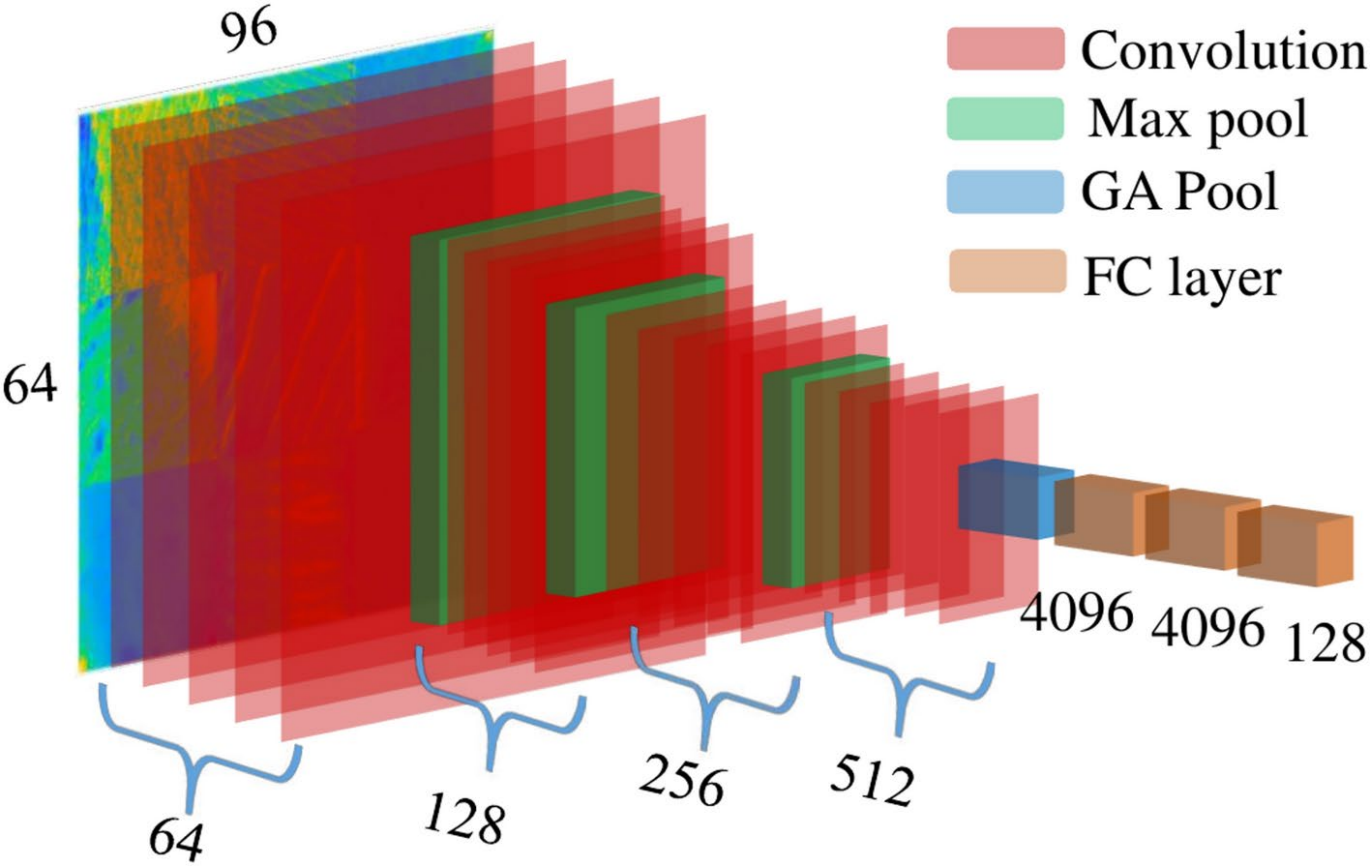
VGGish architecture.

**1. VGGish: **The VGGish architecture, introduced in 2017^57^, was designed for large-scale audio classification tasks. This model was trained on the Youtube-100M dataset, which comprises 5.24 million hours of videos. To process the audio data, it undergoes segmentation into non-overlapping frames of 960 milliseconds. Subsequently, two dimensional 96 $\times$ 64 log-mel spectrograms are generated via a time-frequency conversion process using the STFT and 64 mel-spaced frequency bins integration^58^. The VGGish architecture consisting of 62 million weights, illustrated in Figure 7.

**Fig. 8:**
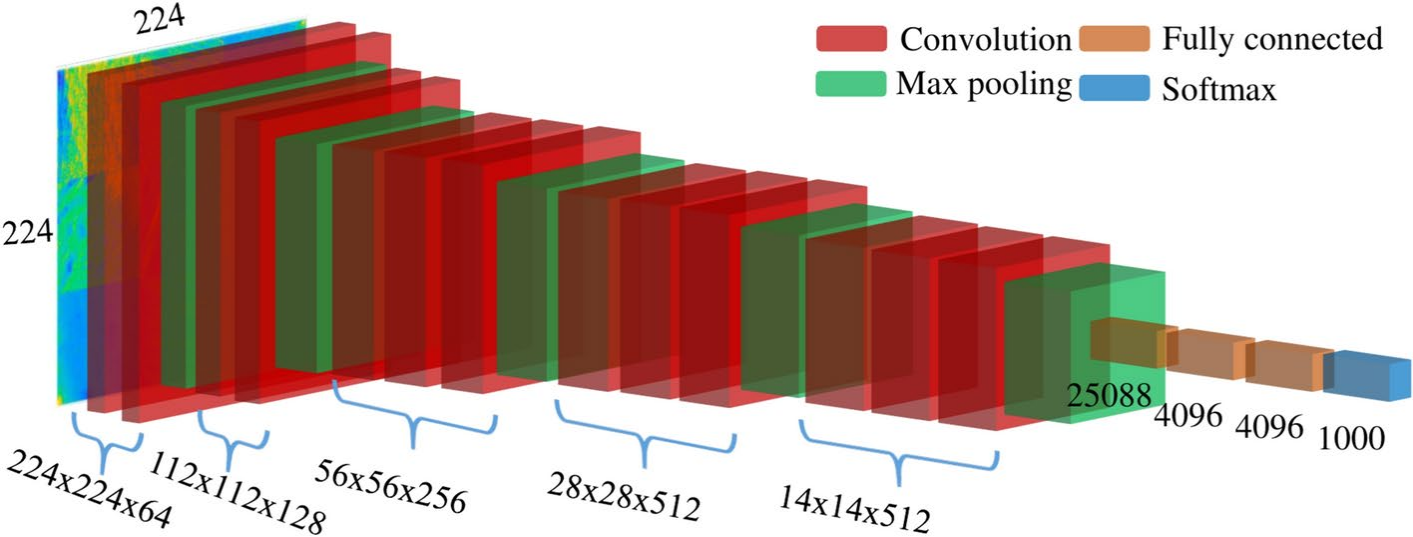
VGG16 architecture.

**2. VGG-16:** The VGG architecture, proposed by Visual Geometry Group, achieved first leading position in the localization and the second position in the classification in ILSVRC 2014. Notably, the VGG16 variant of this architecture is characterized by a composition of 13 convolutional layers wherein small 3x3 kernels and 2x2 polling layers are employed, followed by 3 fully connected layers. The use of small size kernels helped to increase the depth of the network which has 138 million parameters^59^. The VGG-16 architecture is depicted in Figure 8.

**Fig. 9:**
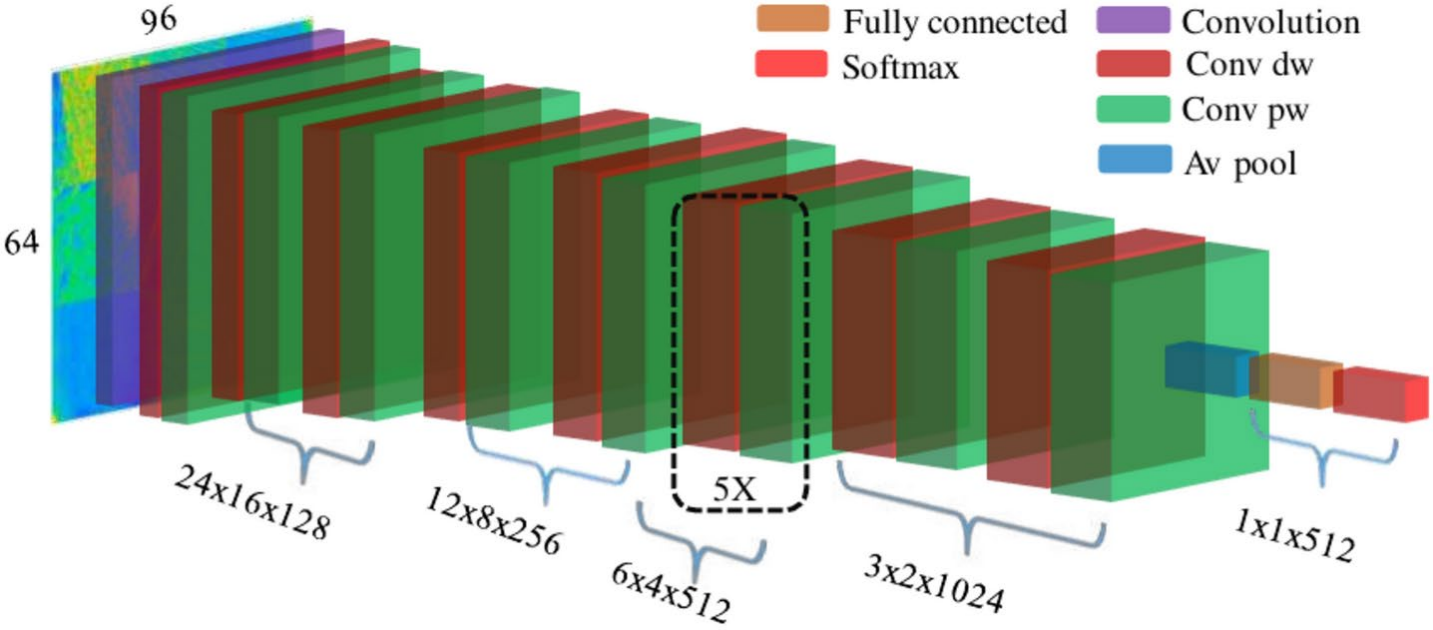
Yamnet architecture.

**3. YamNet: **YamNet (Yet Another Mobile Network) is a pretrained deep neural network developed for audio analysis tasks, based on the MobileNetV1 depthwise-separable convolution architecture^60,61^. As shown in Figure 9, YamNet comprising 27 convolutional layers, each followed by a ReLU activation layer and a batch normalization layer, as well as average pooling layers, fully connected layers, softmax layer, and a final classification layer. YamNet is trained on the Audio Set YouTube corpus^62^ to predict 521 different audio event categories. It processes an input mel spectrogram with dimensions of (48, 32, 32)^63^.

#### Proposed MBH-LPQ model features

**Fig. 10:**
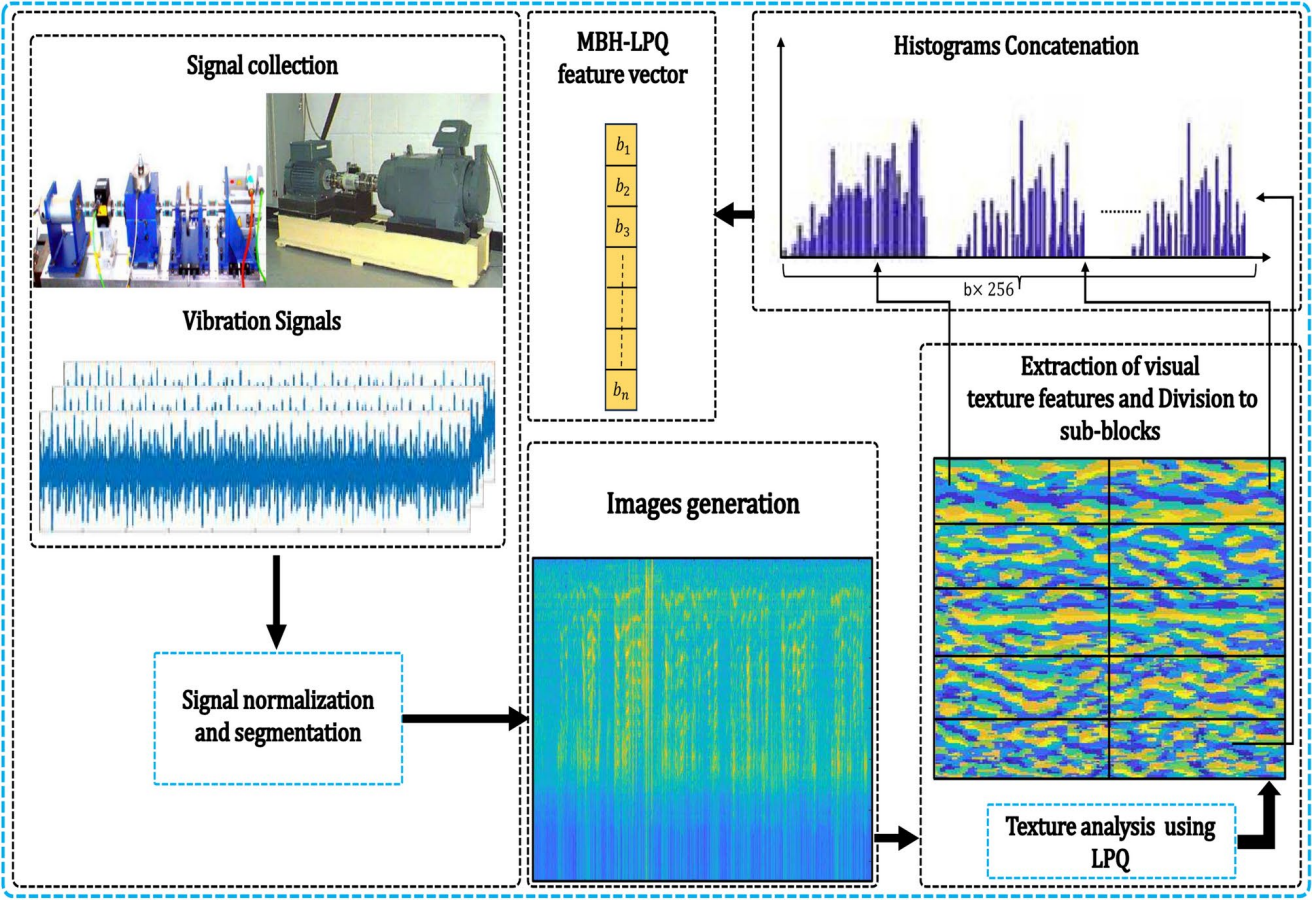
Proposed MBH-LPQ features extraction model.

Our work focuses on developing a reliable system for diagnosing various bearing faults using shallow features. As shown in Figure 10, the proposed framework consists of several key steps. First, We preprocessed the original vibration waveforms by normalizing and splitting them into samples. The next step involves generating a log Mel-spectrogram image from the processed signal. We then analyze the visual texture of this spectrogram using the Local Phase Quantization (LPQ) method. Following LPQ analysis, we divide the LPQ-processed image into $b$ non-overlapping sub-blocks. For each sub-block, we compute a 256-bin histogram that captures the local texture information. These histograms are then concatenated to form a single feature vector of size $b \times 256$. The final step involves aggregating these feature vectors to create the MBH-LPQ descriptor, which encapsulates the texture information of the entire image. The mathematical steps of this process are detailed in Algorithm 1.

**Algorithm 1:**
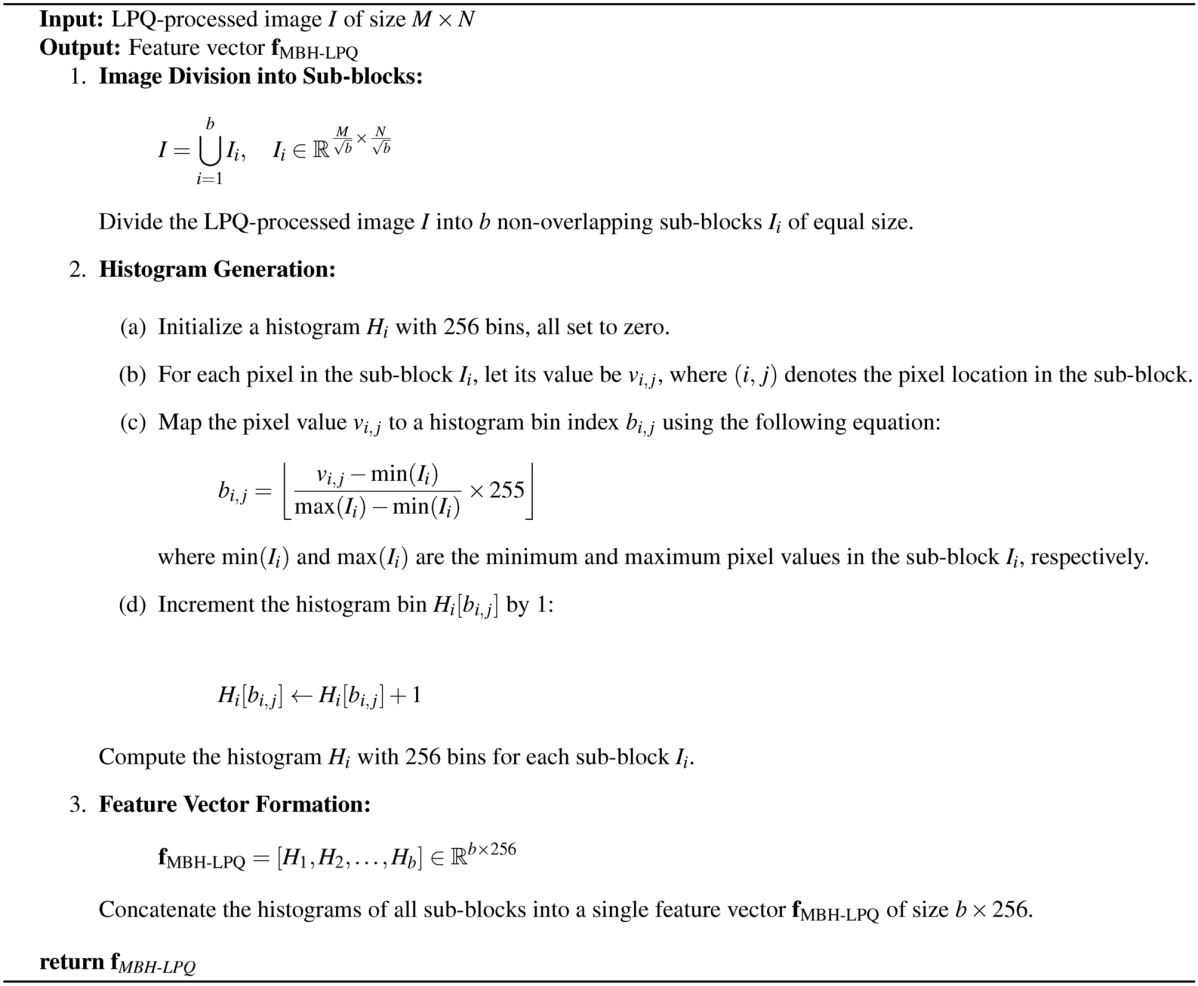
Proposed MBH-LPQ Model Features.

### Linear subspace projection and dimensionality reduction

After extracting features (both deep and shallow features) and concatenating histograms of each block into a vector for our proposed descriptor MBH-LPQ, which represents the characteristics of the spectrogram images, we apply principal component analysis (PCA) to reduce the dimensionality and then apply the exponential discriminant analysis (EDA) algorithm for dimensionality reduction, feature projection, and classification of these vectors. EDA, a robust supervised technique introduced by Zhang et al.^64^, is an exponential extension of Linear Discriminant Analysis (LDA)^65^. The primary objective of this method is to minimize intra-class variability while maximizing inter-class variability, thus enhancing the discriminative power of the features. The transformation from LDA to EDA is accomplished by introducing an exponential function to the LDA equation, as illustrated in equation 14. This modification, as detailed in equation 18, has demonstrated significant improvements in several studies^66,67^.

14 $$\begin{aligned} S_b \textbf{v} = \lambda S_w \end{aligned}$$

In the above equation, $S_b$ and $S_w$ denote the inter-class and intra-class variability matrices, respectively. These matrices are mathematically defined as:

15 $$\begin{aligned} S_b= & \sum _{s=1}^{S} (\textbf{w}_s - \bar{\textbf{w}})(\textbf{w}_s - \bar{\textbf{w}})^T \end{aligned}$$

16 $$\begin{aligned} S_w= & \sum _{s=1}^{S} \frac{1}{n_s} \sum _{i=1}^{n_s} (\textbf{w}_i^s - \bar{\textbf{w}}_s)(\textbf{w}_i^s - \bar{\textbf{w}}_s)^T \end{aligned}$$

Here, $s$ represents the class, $n_s$ is the number of feature vectors $\textbf{w}_s$ within each class $s$, and $\bar{\textbf{w}}_s$ is the mean feature vector for each class, calculated as:

17 $$\begin{aligned} \bar{\textbf{W}}_s = \frac{1}{n_s} \sum _{i=1}^{n_s} \textbf{W}_i^s \end{aligned}$$

Applying the exponential function to both sides of the LDA equation results in the EDA method, as shown below:

18 $$\begin{aligned} \exp (S_b) \textbf{v} = \lambda \exp (S_w) \end{aligned}$$

### Matching and fusion

After dimensionality reduction, the feature vector undergoes a matching process utilizing the cosine distance metric within a discriminant subspace, as defined in Equation 19.

19 $$\begin{aligned} \cos \left( \textbf{V}_{t_1}, \textbf{V}_{t_2}\right) = \frac{\textbf{V}_{t_1}^T \cdot \textbf{V}_{t_2}}{\Vert \textbf{V}_{t_1}\Vert \cdot \Vert \textbf{V}_{t_2}\Vert } \end{aligned}$$

Here, $\textbf{V}_{t_1}$ and $\textbf{V}_{t_2}$ represent vectors, $\textbf{V}_{t_1} \cdot \textbf{V}_{t_2}$ is the dot product of vectors $\textbf{V}_{t_1}$ and $\textbf{V}_{t_2}$, and $\Vert \textbf{V}_{t_1}\Vert$ and $\Vert \textbf{V}_{t_2}\Vert$ are the magnitudes of vectors $\textbf{V}_{t_1}$ and $\textbf{V}_{t_2}$, respectively.

This metric is highly effective in comparing feature vectors, highlighting the discriminative enhancement achieved through the integration of the EDA algorithm. In our Bearing Fault Diagnosis framework, we employ the Weighted Sum (WS) fusion technique to combine deep and shallow features^68^. The WS method is chosen for its demonstrated ability to enhance system performance by strategically merging the two types of features^69^. This approach capitalizes on the unique strengths of both deep and shallow features, significantly improving the accuracy of our diagnostic system. The formula for WS fusion is presented as follows:

20 $$\begin{aligned} WS = \sum _{i=1}^{K} w_{i} \cdot s_{i} \end{aligned}$$

where $WS$ denotes the aggregated weighted sum, $K$ represents the total number of inputs, $w_{i}$ is the weight assigned to the $i$-th input, and $s_{i}$ corresponds to the value or measurement derived from the $i$-th input.

## Experimental setup

This section details the experimental procedures carried out on two datasets: the CWRU dataset and the PU dataset. First, we provide a comprehensive description of these datasets. Following this, we outline the protocols employed during both the training and testing phases, including the various parameter settings used. These parameters encompass the conversion of continuous vibrational waveforms to images and the subsequent feature extraction stages. Finally, we present the experimental results, accompanied by discussions and comparisons with existing methods. All experiments were conducted on an HP laptop running Windows 10 64-bit, powered by an Intel(R) Core(TM) i7-6600U CPU @ 2.60GHz (4 cores) with 16 GB of RAM. MATLAB R2021a was used for data processing and the development of the neural network models.

### Datasets description

In this section, we conducted extensive evaluation experiments using two popular bearing benchmark datasets, namely the Case Western Reserve University (CWRU)^27^ and Paderborn University (PU)^28^ datasets, to verify the effectiveness of our proposed fault diagnosis approach. These datasets include artificially generated and real defect data with varying damage levels, methods, diameters, and health statuses. The details of these datasets are presented below:

#### CWRU dataset

**Fig. 11:**
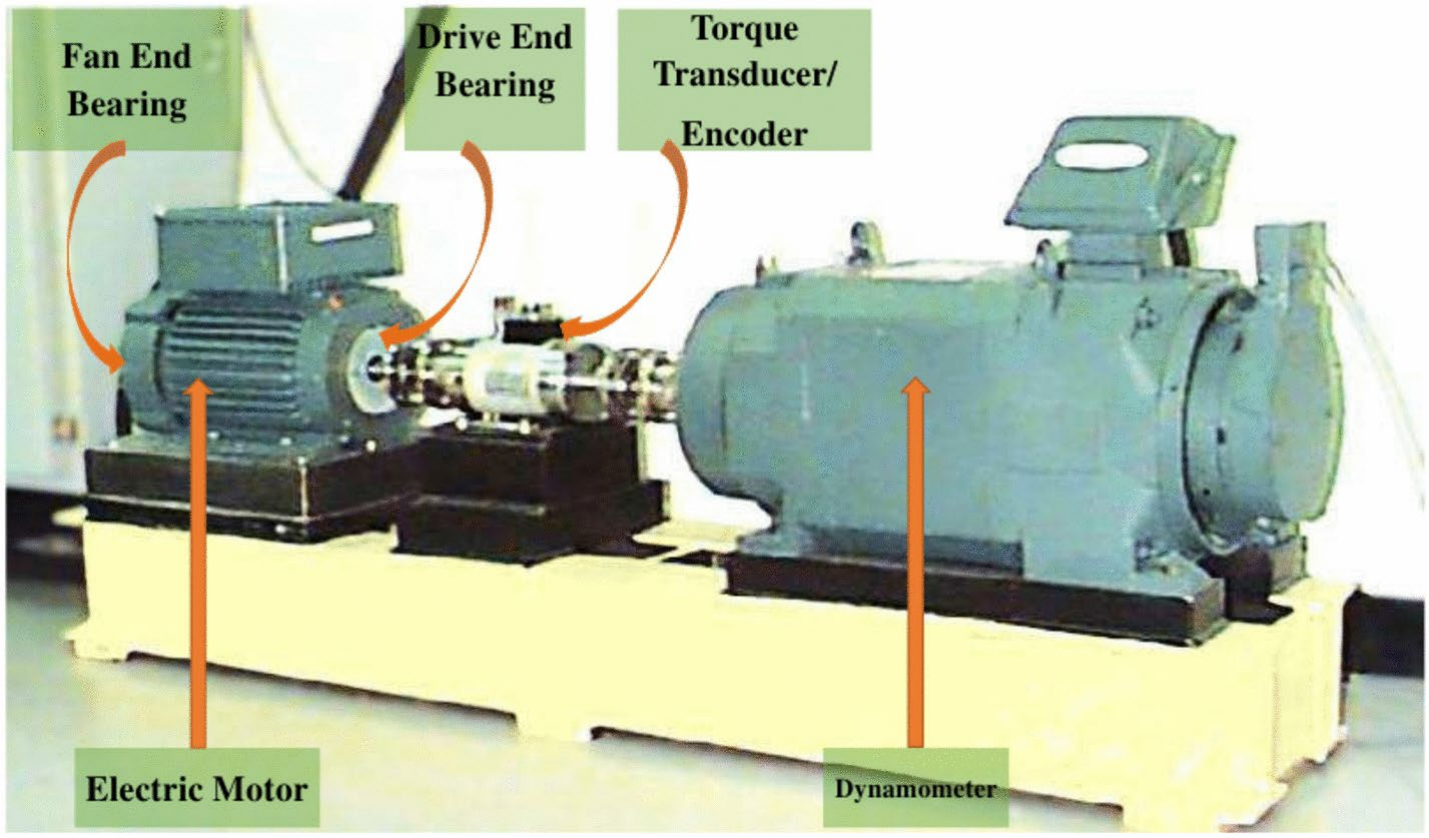
Test Rig of CWRU Dataset.

In the CWRU Bearing Dataset, experiments were conducted using a 2-hp Reliance Electric motor, as depicted in Figure 11. Single-point faults were introduced to the test bearings using electro-discharge machining. Fault diameters of 0.007, 0.014, 0.021, 0.028, and 0.04 inches were introduced separately at the inner race, ball, and outer race. Accelerometers were utilized to gather vibration data, positioned at the 12 o’clock position on both the drive end and fan end of the motor housing. Since outer race faults are fixed faults, accelerometers were also used at the 3, 6, and 12 o’clock positions. Data were collected digitally at a rate of 12,000 samples per second.

Additionally, data was gathered at a rate of 48,000 samples per second specifically for detecting faults in the drive end bearing. The vibration signals were acquired utilizing a 16-channel DAT recorder. Data on speed and horsepower were collected through a torque transducer/encoder and were manually recorded. For faults with diameters of 7, 14, and 21 mils, SKF bearings were employed, while NTN equivalent bearings were used for faults measuring 28 mils and 40 mils, selected based on operational conditions and fault sizes. In this study, fault conditions are classified into nine groups, resulting in a dataset consisting of ten classes: one for normal operation and nine for faulty conditions. Signals collected at a sampling frequency of 48,000 samples per second were considered, under a condition of 1772 rpm with a load of 1 horsepower (hp). The details of the data used are presented in Table 1.

**Table 1:** Description of classes size and labels (CWRU dataset).

Labels	Condition	Fault diameter (inches)	Training samples	Test samples
1	H	-	80	20
2		0.007	80	20
3	IRF	0.014	80	20
4		0.021	80	20
5		0.007	80	20
6	BF	0.014	80	20
7		0.021	80	20
8		0.007	80	20
9	ORF	0.014	80	20
10		0.021	80	20
Total samples			**800**	**200**
	H:Healthy IRF:Inner Race Fault BF:Ball Fault ORF:Outer Race Fault			

#### PU Dataset

**Fig. 12:**
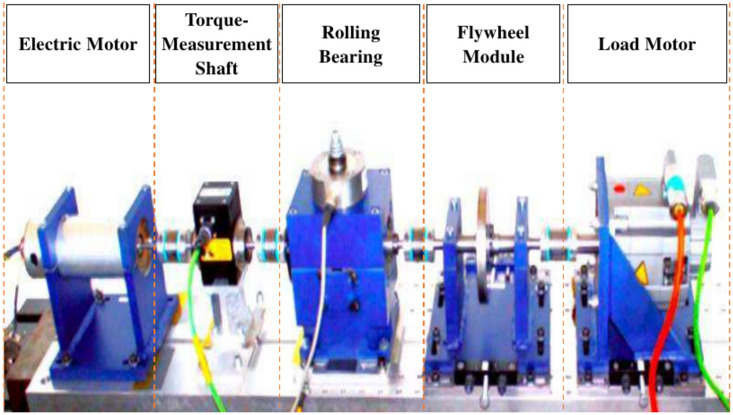
Test Rig of PU Dataset.

In the Paderborn University (PU) bearing dataset, the experimental bearing test setup consists of an electric motor, a torque measurement shaft, a rolling bearing test module, a flywheel, and a load motor, as shown in Figure 12. The test rig utilizes a 6203 bearing operating under constant conditions. The vibration signals were captured by measuring the acceleration of the bearing housing at the adapter located at the upper extremity of the rolling bearing module, with a sampling rate of 64,000 samples per second. This dataset encompasses data of 32 experimental bearings, categorized into three distinct groups: healthy state, artificially induced defects, and real defects generated to simulate accelerated lifetime conditions. For the purpose of this study, the dataset is divided into ten classes: one representing healthy conditions and nine representing defective conditions. The details of the operating settings and descriptions of the data used are presented in Tables 2 and 3, respectively.

**Table 2:** Operating settings.

Name of Setting	Load Torque(Nm)	Radial Load (N)	Rotational speed (rpm)
N15_M07_F10	0.7	1000	1500

**Table 3:** Description of classes size and labels (PU dataset).

Labels	Condition	Bearing code	Damage Method	Training samples	Test samples
1	H	K001	-	272	68
2	IRF	KI01	EDM	272	68
3	IRF	KI03	EE	272	68
4	IRF	KI07	EE	272	68
5	IRF	KI04	P	272	68
6	ORF	KA01	EDM	272	68
7	ORF	KA03	EE	272	68
8	ORF	KA05	EE	272	68
9	ORF	KA04	P	272	68
10	ORF+IRF	KB23	EDM	272	68
Total samples				**2720**	**680**
	EDM:Electrical Discharge Machining EE:Electric Engraver P:Pitting				

### Protocol and parameters settings

In this paper, for the CWRU dataset, a total dataset consisting of ten classes was prepared, one for normal operation and nine for faulty conditions. Details of the description of the data used are provided in Table 1. To evaluate the proposed approach, each continuous vibrational waveforms is divided into input parts containing 100 samples. Each sample containing 4710 data points, resulting in a total of 1,000 samples across the 10 classes. Although training size plays a crucial role in diagnostic performance, as highlighted in^70^, this study adopts an 80/20 split for both datasets, following standard benchmark practices to ensure a fair comparison with existing works. Specifically, for the CWRU dataset, 800 samples were allocated for training and 200 for testing. As for the PU dataset, which also consists of ten classes: one class represents the healthy state and nine represent the faulty state. Each class consists of 17 bearing vibration signals. These signals were divided into 340 samples per class, with each sample containing 12,500 data points. During the preparation phase, we use random partitioning to divide the dataset into training and test sets. 80% of the samples are randomly selected for the training set, while the remaining 20% are reserved for testing. Details of the operating settings and descriptions of the data used are presented in Tables 2 and 3, respectively. The vibration waveforms are transformed into Log-Mel spectrogram images, which provide a three-dimensional representation of the signal. The horizontal axis represents time variation, the vertical axis displays the frequency spectrum, and the intensity of each point in the image reflects the amplitude of the signal. In our study, spectrograms are generated using a Hamming window function with a 50% overlap between frames, preventing information loss at the edges. The Fast Fourier Transform (FFT) is computed with a window size of 512 samples. After converting the vibration waveforms into Log-Mel spectrograms, we then extract four distinct shallow texture features using various local descriptors: LBP, LDP, basic LPQ, and our newly developed descriptor, MBH-LPQ. Additionally, we extracted three deep features using CNN models. To ensure a fair comparison among the shallow descriptors, we maintain consistent parameters with those used for the proposed MBH-LPQ descriptor. We did not subdivide the images, preprocess them, or compute histograms. Instead, we vectorized each component into a single feature vector. For the MBH-LPQ descriptor, we conducted experiments to optimize its performance by exploring various combinations of the three scales generated by basic LPQ, thereby extracting the descriptor across different scales. The window size parameter R for basic LPQ was adjusted, selecting values of R from the set {3, 5, 7}. The spectrogram image was processed with LPQ and then divided into 10 blocks. The histograms from each block were concatenated to form a feature vector, as illustrated in Figure 2. The resulting vectors from the three scales were combined to create a feature vector with dimensions of 1 $\times$ 2560 for each scale. For the deep features, we utilized three pre-trained CNN models: VGG-16, YamNet, and VGGish. Features were extracted from specific layers of each model: for VGG-16, from “fc6”, “fc7”, and “fc8”; for YamNet, from “dense”, and for VGGish, from “fc1_1”,“fc1_2”, and “fc2”. Regarding the WS fusion method, after numerous experiments and varying the weights from 0.1 to 0.9 in increments of 0.1, the final weights were set to 0.8 for the VGGish model and 0.2 for the proposed MBH-LPQ model to achieve the highest possible classification accuracy.

### Results and discussions

The experimental results of our study on the CWRU and PU datasets are detailed in Tables 4 and 5, offering a comprehensive analysis of various experiments designed to assess the performance of the proposed VGGish+MBH-LPQ method. They summarize the performance of the diagnosis models using traditional shallow descriptors such as LBP, LDP, and basic LPQ. Notably, these tables include the basic LPQ descriptor results at different scales (3, 5, and 7). To enable a direct performance comparison, we also introduce the proposed MBH-LPQ descriptor, evaluated at the same scales as basic LPQ. Further, we assess the performance of deep features extracted from various CNN models, including VGG-16, VGGish, and YamNet, with accuracy metrics reported for different output layers to evaluate their individual contributions. The tables also report the percentage of accuracy obtained by fusing the scores of the best accuracy obtained by the shallow description (MBH-LPQ) with the top CNN model result (VGGish), using weighted sum (WS) fusion.

Table 6 presents the accuracy achieved by the proposed MBH-LPQ descriptor, with the number of sub-blocks ranging from 1 to 12. This parameter was varied across both datasets to identify the optimal number of sub-blocks, with the results clearly illustrating the impact of image subdivision and histogram extraction on classification accuracy. Table 7 lists the different combination ratios of the weighted sum fusion of the proposed MBH-LPQ descriptor and VGGish method.

**Table 4:** Results of experiments using shallow features and deep features on the CWRU dataset.

Features Type	Descriptor	Scales and Layers	**Accuracy(%)**
Shallow Features	LBP	/	92.11
	LDP	/	87.89
	LPQ	R=3	84.21
		R=5	86.32
		R=7	87.37
	**MBH-LPQ**	R=3	94.21
		R=5	97.37
		**R=7**	**97.89**
Deep Features	VGG16	fc6	96.84
		fc7	90.53
		fc8	72.63
	YamNet	dense	71.58
	**VGGish**	**fc1_1**	**98.42**
		fc1_2	92.11
		fc2	71.05
**Fusion**	**VGGish+MBH-LPQ**	**fc1_1+(R=7)**	**98.95**

**Table 5:** Results of experiments using shallow features and deep features on the PADERBORN dataset.

Features Type	Descriptor	Scales and Layers	**Accuracy(%)**
Shallow Features	LBP	/	77.91
	LDP	/	76.27
	LPQ	R=3	79.25
		R=5	72.54
		R=7	84.48
	**MBH-LPQ**	R=3	94.33
		R=5	94.93
		**R=7**	**97.01**
Deep Features	VGG16	fc6	94.48
		fc7	91.79
		fc8	85.97
	YamNet	dense	60.00
	**VGGish**	**fc1_1**	**99.10**
		fc1_2	94.63
		fc2	63.73
**Fusion**	**VGGish+MBH-LPQ**	**fc1_1+(R=7)**	**100**

#### Selecting the number of sub-blocks

Table 6 demonstrates the impact of varying the number of sub-blocks on the performance of our system using the proposed MBH LPQ descriptor. In this study, the selection of the number of sub-blocks, denoted as ’*b*’ was determined through empirical analysis. The value of ’*b*’ was systematically varied, ranging from 1 to 12 in increments of 2. The optimal configuration, which delivered the highest performance, consisted of 10 sub-blocks, achieving accuracy rates of 97.89% and 97.01% for the CWRU and PU datasets, respectively.

**Table 6:** The effect of the number of sub-blocks of the proposed MBH-LPQ descriptor.

Features	Number of sub-blocks (*b*)	CWRU dataset	PU dataset
		Accuracy (%)	Accuracy (%)
MBH-LPQ(R=7)	1	87.37	84.48
	2	93.68	90.75
	4	87.89	86.57
	6	97.37	91.49
	8	95.26	89.55
	**10**	**97.89**	**97.01**
	12	99.47	94.93

#### Impact of shallow texture descriptors

**Fig. 13:**
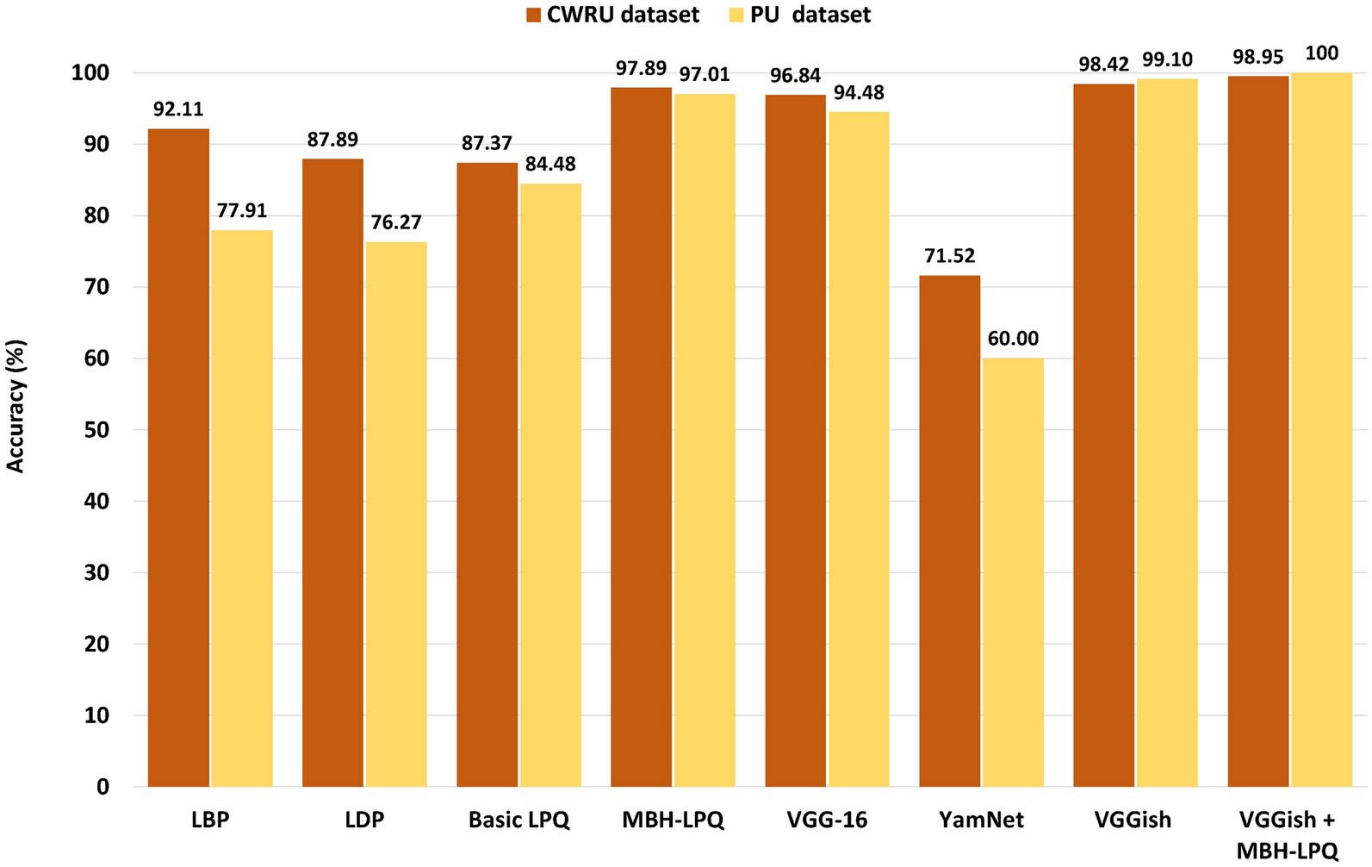
Classification accuracy of different models.

Shallow descriptors have been widely used across various fields in prior research, particularly for feature extraction in image recognition. The effectiveness of these methods can vary depending on the specific application. In this study, three distinct descriptors LBP, LDP, and basic LPQ were evaluated for their performance in classifying load signals and identifying fault types in rolling bearings. Figure 13 and Tables 4 and 5 detailing the accuracy rates obtained from these descriptors. For the CWRU dataset, LBP, LDP, and basic LPQ achieved accuracy rates of 92.11%, 87.89%, and 87.37%, respectively. In comparison, the PU dataset yielded average accuracy rates of 77.91%, 76.27%, and 84.48%, respectively. Notably, the basic LPQ descriptor demonstrated superior performance in the PU dataset, surpassing LBP by 6.57% and LDP by 8.21%. However, in the CWRU dataset, LBP outperformed LDP and basic LPQ by 4.22% and 4.74%, respectively. These findings underscore the effectiveness of shallow texture descriptors, particularly the LPQ descriptor, in extracting meaningful features from spectrogram images for fault diagnosis and classification.

#### Impact of deep learning methods

Deep learning has proven to be an effective approach for extracting and recognizing complex features, significantly enhancing fault diagnosis and classification tasks. In this study, we evaluated the performance of three pre-trained models YamNet, VGGish, and VGG16 using bearing vibration signals. The results, appear in Figure 13 and detailed in Tables 4 and 5, show that the VGG16 model achieved accuracy rates ranging from 72.63% to 96.84% for the CWRU dataset and from 85.97% to 94.48% for the PU dataset. The VGGish model, pre-trained on audio data, demonstrated even higher accuracy, with rates of 98.42% and 99.10% for the CWRU and PU datasets, respectively. In contrast, the YamNet model exhibited lower performance, with accuracy rates of 71.58% and 60.00% for the CWRU and PU datasets, respectively. These findings suggest that while the VGGish and VGG16 models are highly effective for vibration signal analysis, particularly VGGish for audio-based data, the YamNet model may be less suitable for this specific application.

#### Benefits of MBH-LPQ model features

The MBH-LPQ approach demonstrates a significant improvement in feature discrimination over the basic LPQ method and other shallow texture descriptors, as evidenced by its performance on the CWRU and PU datasets (see Tables 4 and 5). Specifically, MBH-LPQ increased accuracy by 10.52% on the CWRU dataset (from 87.37% to 97.89%) and by 12.53% on the PU dataset (from 84.48% to 97.01%) compared to the basic LPQ method. This enhancement is attributed to the method’s effective partitioning of Log Mel-spectrogram images into sub-blocks and the subsequent representation of localized regions through histograms. The MBH-LPQ descriptor excels in capturing essential texture information, thereby outperforming the basic LPQ method. The main strength of MBH-LPQ lies in its ability to deliver substantial accuracy improvements by leveraging detailed localized feature extraction.

#### The power of WS fusion at the score-level stage

**Fig. 14:**
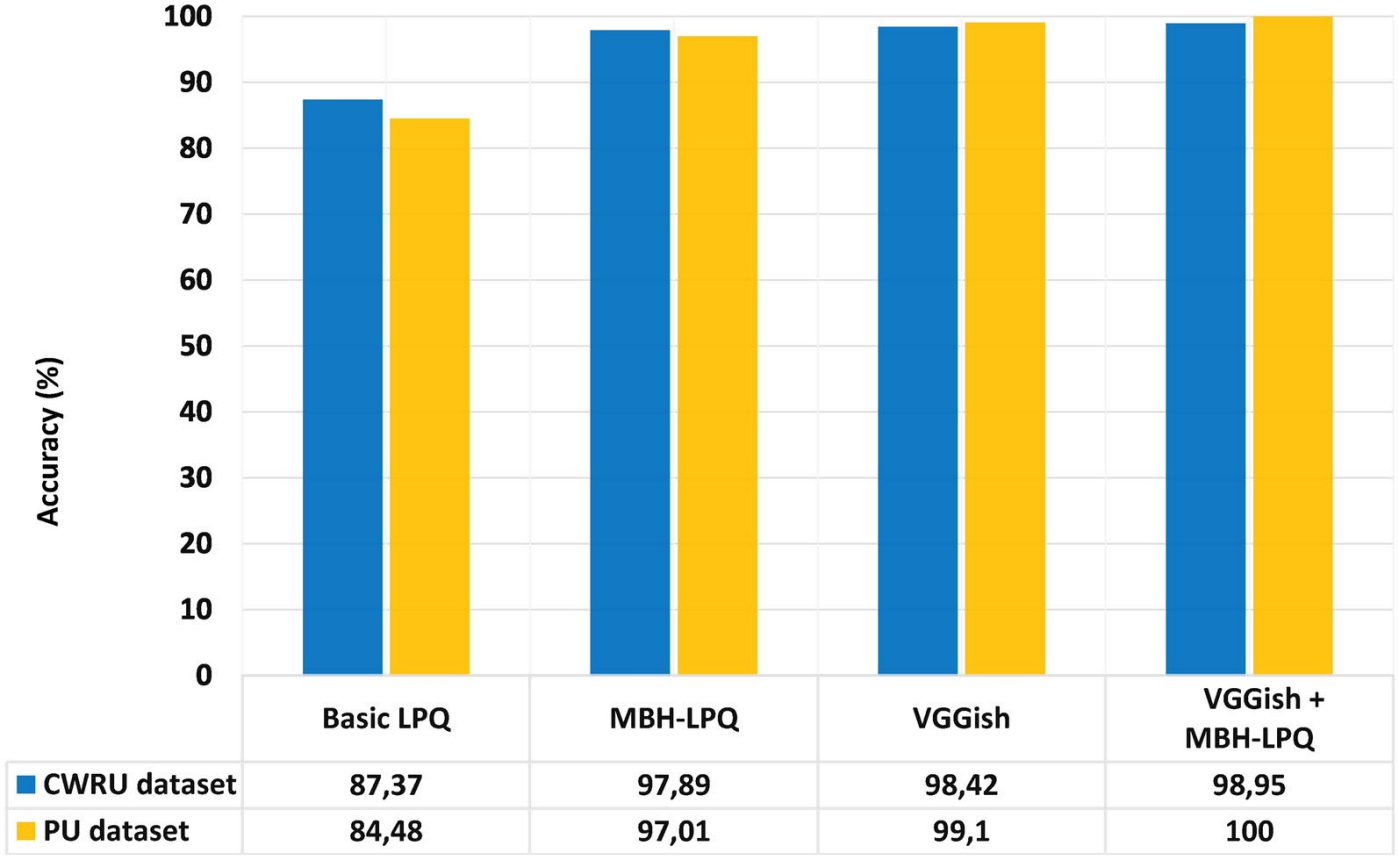
The power of MBH-LPQ and WS fusion on the accuracy level.

We achieved accuracy rates of 97.89% and 97.01% using the proposed shallow MBH-LPQ approach on the CWRU and PU datasets, respectively. Additionally, the VGGish model outperformed other deep learning models, such as VGG-16 and Yamnet, achieving the highest accuracy rates of 98.42% on the CWRU dataset and 99.10% on the PU dataset. To further enhance diagnostic performance, we combined the proposed shallow MBH-LPQ approach with the VGGish deep model using a Weighted Sum (WS) fusion method at the score level. A series of experiments was conducted to determine the optimal weight ratio for the WS fusion method. Specifically, the weights were adjusted within a range of 0.1 to 0.9 in increments of 0.1 for both the VGGish model and the MBH-LPQ model. After extensive testing, the final weights were set to 0.8 for the VGGish model and 0.2 for the MBH-LPQ model. These selected weights highlight the complementary contributions of both models, with the VGGish model providing robust high-level feature representations and the MBH-LPQ model contributing critical texture-based information. By leveraging this WS fusion method, the diagnostic accuracy was further improved, achieving remarkable rates of 98.95% for the CWRU dataset and a perfect 100% for the PU dataset, as depicted in Figure 14 and Table 7. This underscores the effectiveness of combining deep learning techniques with texture-based descriptors for classifying different bearing faults.

**Table 7:** The impact of WS ratio on weighted sum fusion of the proposed MBH-LPQ-VGGish method.

MBH-LPQ	VGGish	CWRU dataset	PU dataset
(WS ratio (%))	(WS ratio (%))	Accuracy (%)	Accuracy (%)
10	90	98.01	97.16
20	80	98.16	97.31
30	70	98.34	97.46
40	60	98.34	97.76
50	50	98.45	97.91
60	40	98.77	98.21
70	30	98.95	98.81
**80**	**20**	**98**.**95**	**100**
90	10	98.95	99.40

**Fig. 15:**
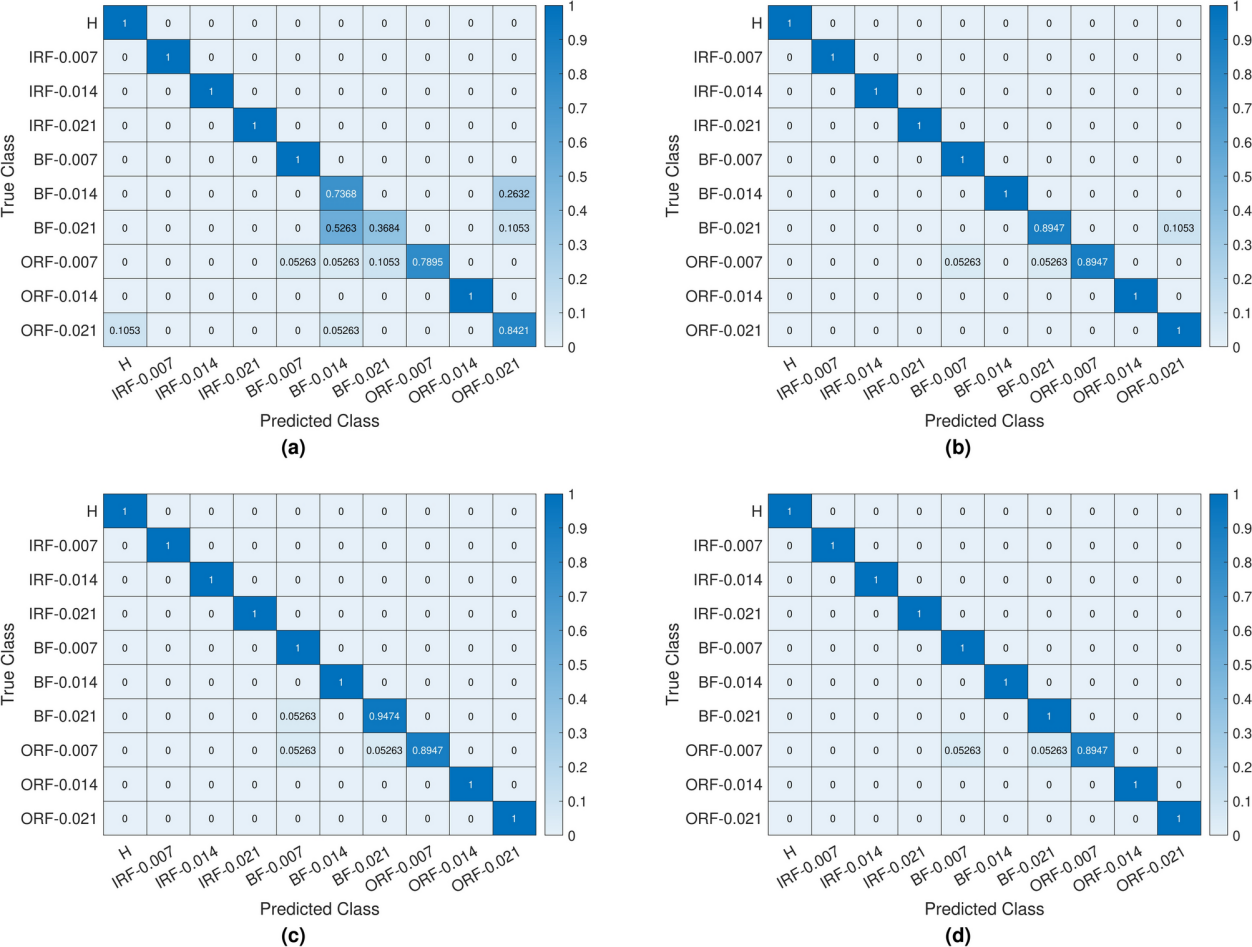
The Confusion matrices of the test results of CWRU dataset): (a) Basic LPQ , (b) MBH-LPQ ,(c) VGGish , (d) Fusion(VGGish + MBH-LPQ ).

**Fig. 16:**
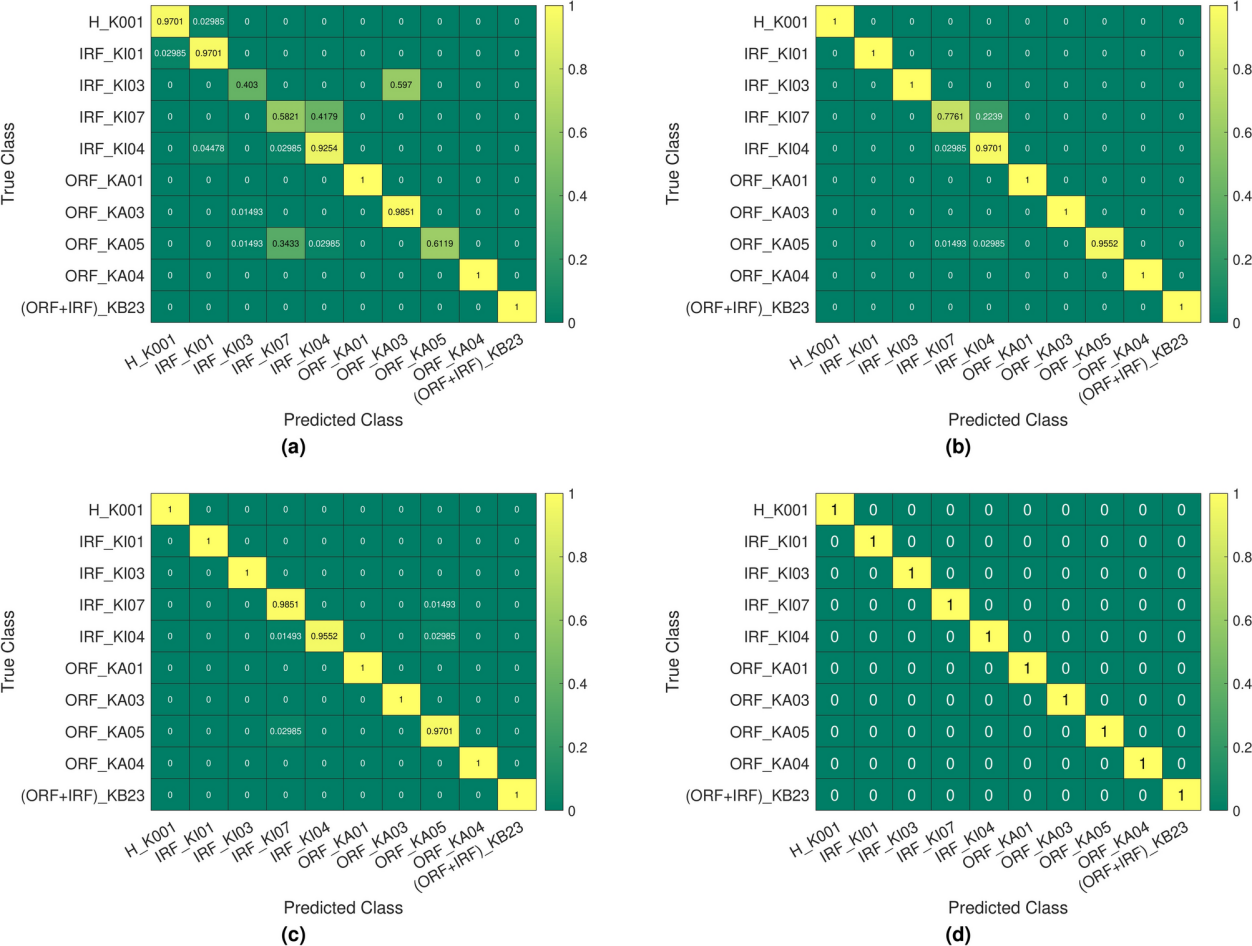
The Confusion matrices of the test results of (PU dataset ): (a) Basic LPQ , (b) MBH-LPQ ,(c) VGGish ,(d) Fusion(VGGish + MBH-LPQ).

The confusion matrices of our proposed models for the CWRU dataset are presented in Figure 15 to illustrate the prediction results of the methods explored in this study. In the fault classification results using the basic LPQ method, 26.32% of the 0.014 inches Ball Fault are incorrectly identified as 0.021 inches Outer Race Fault. Additionally, 52.63% of the 0.021 inches Ball Fault are misclassified as 0.014 inches Ball Fault, and 10.53% are misclassified as 0.021 inches Outer Race Fault. For the 0.007-inch Outer Race Fault, 5.26% of the 0.007 inches Outer Race Fault are incorrectly identified as 0.007 inches Ball Fault, 5.26% and 10.53% were incorrectly classified as a 0.014” and 0.021” inches Ball Fault, respectively. 10.53% of the 0.021 inches Outer Race Fault are incorrectly identified as a health condition, and 5.26% are misclassified as 0.014 inches Ball Fault. Accordingly, the misclassified classes are scattered within the confusion matrix (see Figure 15a). It is clear that the proposed new MBH-LPQ descriptor performed effectively, accurately classifying eight classes out of ten, 10.53% of the 0.021 inches Ball Fault are incorrectly identified as 0.021 inches Outer Race Fault. 5.26% and 5.26% of the 0.007 inches Outer Race Fault are wrongly predicted as 0.007 and 0.021 inches Ball Fault, respectively. this has led to a slight scatter within the confusion matrix (see Figure 15b). In the VGGish deep learning model, 5.26% of the 0.021 inches Ball Fault are misidentified as 0.007 inches Ball Fault, and 5.26% of the 0.007 inches Outer Race Fault are incorrectly identified as 0.007 inches Ball Fault, and 5.26% are incorrectly identified as 0.021 inches Ball Fault and , As observed in the confusion matrix (see Figure 15c), this led to a slight dispersion between the two misclassified classes. However, Our proposed fusion approach VGGish +MBH-LPQ obtains near-fault-free confusion matrix (see Figure 15d). Only 5.26% of the 0.007 inches Outer Race Fault are misidentified as 0.007 inches Ball Fault, and 5.26% were incorrectly identified as 0.021-inch ball fault. For the second dataset (PU), as shown in Figure 16, the confusion matrix were used to illustrate the prediction results of the methods based on our study. Where we can see the faults classification of the basic LPQ method is mainly that 2.99% of the Healthy state (H-K001) are incorrectly identified as Inner Race Fault (IRF-KI01). 2.99% of the Inner Race Fault (IRF-KI01) are incorrectly identified as Healthy state (H-K001). 59.7% of the Inner Race Fault (IRF-KI03) are incorrectly identified as Outer Race Fault (ORF-KA03). 41.79% of the Inner Race Fault (IRF-KI07) are incorrectly identified as Inner Race Fault (IRF-KI04). 4.48% of the Inner Race Fault (IRF-KI04) are incorrectly identified as Inner Race Fault (IRF-KI01), and 2.99% are incorrectly identified as Inner Race Fault (IRF-KI07). 1.49% of the Outer Race Fault (ORF-KA03) are incorrectly identified as Inner Race Fault (IRF-KI03). 1.49% of the Outer Race Fault (ORF-KA05) are incorrectly identified as Inner Race Fault (IRF-KI03), and 34.33% are incorrectly identified as Inner Race Fault (IRF-KI07), and 2.98% are incorrectly identified as Inner Race Fault (IRF-KI04). Accordingly, we observe a dispersion of misclassified classes within the confusion matrix (See Figure 16a). While the classification of bearing faults using the proposed new texture descriptor MBH-LPQ performed well compared to the basic LPQ model, accurately classifying seven classes compared to only three classes for the basic LPQ model, according to the confusion matrix (see Figure 16b), we observe that 22.39% of the Inner Race Fault (IRF-KI07) are incorrectly identified as Inner Race Fault (IRF-KI04). 2.99% of the Inner Race Fault (IRF-KI04) are incorrectly identified as Inner Race Fault (IRF-KI07). 1.49% of the Outer Race Fault (ORF-KA05) are incorrectly identified as Inner Race Fault (IRF-KI07), and 2.99% are incorrectly identified as Inner Race Fault (IRF-KI04). The misclassification of the VGGish deep learning model is essentially that 1.49% of the Inner Race Fault (IRF-KI07) are incorrectly identified as Outer Race Fault (ORF-KA05), and just 1.49% of the Inner Race Fault (IRF-KI04) are incorrectly identified as Inner Race Fault (IRF-KI07), and 2.99% are incorrectly identified as Outer Race Fault (ORF-KA05). 2.99% of the Outer Race Fault (ORF-KA05) are incorrectly identified as Inner Race Fault (IRF-KI07). Accordingly, we see through the confusion matrix (see Figure 16c) a slight dispersion of the two incorrectly identified classes. Our proposed fusion approach VGGish + MBH-LPQ using the PU dataset obtained a completely fault-free confusion matrix (see Figure 16d) where we observe 100% correct identification of the ten different classes and hence we observe completely defined fault classes.

#### Experimental results under noisy conditions

To further evaluate the the performance of the proposed diagnosis models,and extra Gaussian white noises with varying signal-to-noise ratios (SNRs) of -6 dB, -3 dB, 0 dB,, 3 dB, and 6 dB are added to the original vibration signal. The SNR is mathematically defined by the following formula:

21 $$\begin{aligned} SNR_{\text{( }db)} = 10 \cdot \log \left( \frac{P_{S}}{P_{N}}\right) \end{aligned}$$

Where, ${P_{S}}$ represents the power of the signal, while ${P_{N}}$ denotes the power of the noise. A higher SNR value indicates superior signal quality, signifying reduced interference from noise and a clearer representation of the signal. The CWRU and PU datasets are contaminated by different noise levels to assess the reliability of the proposed method in classifying various motor bearing faults under such circumstances. The specified noise levels serve to validate the accuracy of the proposed approach in comparison to other existing methods for motor bearing fault classification. The performance evaluation and comparison of the proposed models are shown in Table ref. In general, the accuracy of the proposed models MBH-LPQ, VGGish, and VGGish+MBH-LPQ decreased as the S/P ratio increased to -6 dB. Conversely, as the S/P ratio increased above the S/P ratio and approached 6 dB, the model accuracy tended to increase. In conclusion, the bearing data containing noise can interfere with the diagnosis of various bearing faults. However, by comparing with the noiseless MBH-LPQ, VGGish, and VGGish+MBH-LPQ models, it is worth noting that our proposed approach VGGish+MBH-LPQ consistently achieved diagnostic accuracy above 92.11% in all SNR conditions for the CWRU dataset, while for the PU dataset, a high diagnostic accuracy of above 99.25% was achieved, demonstrating unparalleled robustness and noise resistance, both in low SNR environments with strong noise and high SNR scenarios. This noise-based test is specifically designed to evaluate the robustness of the ability of the proposed diagnosis model to maintain performance despite the presence of noise rather than to assess its generalization capability.

**Table 8:** Effects of noise on fault diagnosis accuracy of the proposed methods.

Dataset	Method	-6(db)	-3(db)	0(db)	3(db)	6(db)	None
CWRU	MBH-LPQ	88.42%	92.63%	94.21%	95.26%	95.79%	**97.89%**
	VGGish	91.58%	94.74%	96.32%	96.84%	97.37%	**98.42%**
	VGGish+MBH-LPQ	92.11%	96.32%	96.32%	97.37%	97.89%	**98.95%**
PU	MBH-LPQ	94.78%	95.52%	96.42%	96.72%	96.87%	**97.01%**
	VGGish	96.12%	96.87%	97.91%	98.36%	98.51%	**99.10%**
	VGGish+MBH-LPQ	**99.25%**	**99.40%**	**99.70%**	**99.85%**	**99.25%**	**100%**

### Comparison to existing methods

To demonstrate the efficiency of our proposed approaches, Table 9 showcases a thorough evaluation showcasing the performance of our proposed models MBH-LPQ, VGGish, and VGGish + MBH-LPQ in comparison with the latest state-of-the-art diagnostic techniques. The comparison table presents a set of proposed models, including MBH-LPQ, VGGish, and VGGish + MBH-LPQ, each designed with the ability to handle 10 classes. These models were trained and tested on the CWRU and PU datasets, and their accuracy ranged from 97.89% for MBH-LPQ and 98.42% for VGGish on the CWRU dataset to 97.01% for MBH-LPQ and 99.10% for VGGish on the PU dataset. In contrast, the state-of-the-art methods mentioned in the table use diverse models and CWRU or PU dataset.

For CWRU dataset, Niyongabo et al.^71^ uses a fine-tuned DenseNet-121 with 10 classes, achieving an average accuracy 98.57%. Makrouf et al.^72^ uses a Stacking Classifier for 10 classes, achieving a higher accuracy of 97.2%. Li et al.^73^ presents a TDANET with 10 classes, achieving 97.69% average accuracy. Wang^42^ presents three models with 10 classes, achieving 88.7% accuracy for SMA-VMD-CNN, 90.3% and for SMA-VMD-LSTM and 94.6% for SMA-VMD-CNN-LSTM. Finally, our proposed methods use 10 classes, where MBH-LPQ achieved 97.89% accuracy and VGGish achieved 98.42% accuracy. Also, the VGGish + MBH-LPQ fusion approche through weighted sum (WS) achieved an excellent diagnosis accuracy of 98.95% for the CWRU datast.

While for the second PU dataset, Toma and Kim^44^, used discrete wavelet transform (DWT) and machine learning techniques to extract features. Using the extracted feature set, the three bearing fault conditions were classified using an extreme gradient boosting (XGBoost) classifier, showing an accuracy of 99.3%. Kaige et al.^74^, proposed an approach that takes into account differences in work environment conditions and contaminated data based on a hierarchical branch convolutional neural network (HB-CNN) scheme, achieving classification accuracy of 99.92% for 3 classes and 99.86% for 5 classes. Tianyu et al.^75^, proposed a multi-source domain information fusion network (MDIFN), and under variable operating conditions, achieved an average accuracy of 95.97%. Qi et al.^76^, proposed three models: 2DCNN, 2DCNN + DCGAN, and TF-DLGAN, where the 2DCNN model achieved an average accuracy of 97%, the 2DCNN + DCGAN model achieved an average accuracy of 98.5%, while the TF-DLGAN model based on a deep convolutional generative adversarial network achieved the highest average accuracy of 99.25%.Finally, our results showed that we achieved 97.01% accuracy for the proposed shallow model MBH-LPQ and the VGGish model achieved an accuracy of 99.10%. We also achieved an excellent diagnosis accuracy of 100% through the VGGish + MBH-LPQ fusion approach.

**Table 9:** Comparison of classification accuracy across with the state-of-the-art models.

Dataset	Number of classes	Author	Year	Method	Accuracy(%)
CWRU	10	Niyongabo et al.^71^	2022	fine-tuned DenseNet-12	98.57
		Makrouf et al.^72^	2023	Stacking Classifier	97.2
		Li et al.^73^	2024	TDANET	97.69
		Wang and Nan^42^	2024	SMA-VMD-CNN	88.7
				SMA-VMD-LSTM	90.3
				SMA-VMD-CNN-LSTM	94.6
		**Proposed**	**2024**	**MBH-LPQ**	**97.89**
				**VGGish**	**98.42**
				**VGGish+MBH-LPQ**	**98.95**
PU	3	Toma and Kim.^44^	2020	DWT + XGBoost	99.3
	3	Kaige et al.^74^	2021	HB-CNN	99.92
	5				99.86
	13	Tianyu et al.^75^	2024	MDIFN	95.97
	10	Qi et al.^76^	2024	2DCNN	97
				2DCNN + DCGAN	98.5
				TF-DLGAN	99.25
		**Proposed**	**2024**	**MBH-LPQ**	**97.01**
				**VGGish**	**99.10**
				**VGGish+MBH-LPQ**	**100**

Based on these comparisons, it is evident that our proposed models effectively extracted critical bearing features, delivering high diagnostic accuracy across both the CWRU and PU datasets. Furthermore, the fusion approach demonstrated exceptional diagnostic accuracy, confirming the superior performance of our models compared to other methods.

### Time Complexity Evaluation

To assess the computational complexity of the proposed method, we measured the CPU time required to execute the algorithm. The experiments were conducted on a system equipped with an Intel(R) Core(TM) i5-8365U CPU (1.60 GHz - 1.90 GHz) and 16 GB of RAM, using MATLAB R2021a. Table 10 presents the CPU execution time (in seconds) of our algorithm across two evaluation datasets. The computation time of our approach, utilizing VGGish+MBH-LPQ features, is approximately 1.998 seconds for the CWRU dataset and 1.795 seconds for the PU dataset. These results highlight that the required verification time is minimal, demonstrating the feasibility and effectiveness of our approach for real-time bearing fault diagnosis applications.

**Table 10:** The computation time (s) of the proposed methods using the two datasets.

Dataset	Methods	Time (s)
CWRU	**MBH-LPQ(Our)**	**0.224**
	**VGGish(Our)**	**1.769**
	**VGGish+MBH-LPQ(Our)**	**1.998**
	OCSSA-VMD-CNN-BiLSTM^43^	8.47
	DTL-Res2Net-CBAM^77^	372
	PSO-tuned XGBoost^78^	127.212
	CBAM-MFFCNN^79^	0.1179
PU	**MBH-LPQ(Our)**	**0.134**
	**VGGish(Our)**	**1.654**
	**VGGish+MBH-LPQ(Our)**	**1.795**

## Conclusion and future works

Shallow recognition techniques can effectively extract valuable information from Log Mel-Spectrogram images related to bearings faults features. In this paper, we proposed a novel approach that incorporates two distinct approaches: the deep learning model VGGish and the newly proposed shallow technique called MBH-LPQ. This incorporation aims to minimize information loss during features extraction and enhance the accuracy of fault diagnosis. The objective of this study extends beyond achieving high classification accuracy, it also includes comprehensive comparison of different texture descriptors as well as the impact of deep learning models pre-trained on voice recognition and those pre-trained on image recognition. This comparison is crucial in demonstrating the impact of these models on the diagnostic accuracy of different bearing faults.

The proposed approach effectively performs detection and classification of bearing faults across two distinct datasets. Various shallow and deep recognition techniques were used, and experimental results obtained indicate the effectiveness of the proposed diagnostic models in distinguishing between different bearing fault features and their superiority over existing established approaches. Additionally, combining texture descriptor shallow techniques with deep learning algorithms shows promise in enhancing the accuracy of fault classification across different bearings. The time-frequency domain based feature selection and extraction proved to be highly efficient, yielding very high accuracy. The Log Mel-Spectrogram extracted from the raw audio signal were particularly useful for differentiating between various bearing faults.

Our future research focus on the optimization of the proposed methodology for bearings fault diagnosis and testing it on broader datasets under different load and operating conditions and under different data split strategies to assess the impact of training size on fault diagnosis performance to ensure its effectiveness while reducing computational load to demonstrate generalizability. Additionally, we will explore the potential applicability of this approach in diverse fields beyond bearing fault diagnosis.

## Data Availability

The datasets CWRU and PU analysed during the current study are available at: ”https://engineering.case.edu/bearingdatacenter/download-data-file” and ”https://mb.uni-paderborn.de/kat/datacenter”, respectively.

## References

[1] Chu, T., Nguyen, T., Yoo, H. & Wang, J. A review of vibration analysis and its applications. *Heliyon* (2024). 10.1016/j.heliyon.2024.e26282 PMC10909639 38439821

[2] Aburakhia, S. A., Myers, R. & Shami, A. A hybrid method for condition monitoring and fault diagnosis of rolling bearings with low system delay. *IEEE Transactions on Instrumentation and Measurement* **71**, 1–13 (2022).

[3] Mian, Z. et al. A literature review of fault diagnosis based on ensemble learning. *Engineering Applications of Artificial Intelligence* **127**, 107357 (2024).

[4] Bianchini, C., Immovilli, F., Cocconcelli, M., Rubini, R. & Bellini, A. Fault detection of linear bearings in brushless ac linear motors by vibration analysis. *IEEE Transactions on Industrial Electronics* **58**, 1684–1694 (2010).

[5] Zhang, P., Du, Y., Habetler, T. G. & Lu, B. A survey of condition monitoring and protection methods for medium-voltage induction motors. *IEEE Transactions on Industry Applications* **47**, 34–46 (2010).

[6] Albrecht, P., Appiarius, J., McCoy, R., Owen, E. & Sharma, D. Assessment of the reliability of motors in utility applications - updated. *IEEE Transactions on Energy Conversion* **EC-1**, 39–46, 10.1109/TEC.1986.4765668 (1986).

[7] Report of large motor reliability survey of industrial and commercial installations, part i. *IEEE Transactions on Industry Applications* **IA-21**, 853–864, 10.1109/TIA.1985.349532 (1985).

[8] Report of large motor reliability survey of industrial and commercial installations, part ii. *IEEE Transactions on Industry Applications* **IA-21**, 865–872, 10.1109/TIA.1985.349533 (1985).

[9] Report of large motor reliability survey of industrial and commercial installations: Part 3. *IEEE Transactions on Industry Applications* **IA-23**, 153–158 (1987).

[10] Chen, J., Lin, C., Yao, B., Yang, L. & Ge, H. Intelligent fault diagnosis of rolling bearings with low-quality data: A feature significance and diversity learning method. *Reliability Engineering & System Safety* **237**, 109343 (2023).

[11] Zuo, L., Xu, F., Zhang, C., Xiahou, T. & Liu, Y. A multi-layer spiking neural network-based approach to bearing fault diagnosis. *Reliability Engineering & System Safety* **225**, 108561 (2022).

[12] Wang, D., Tsui, K.-L. & Miao, Q. Prognostics and health management: A review of vibration based bearing and gear health indicators. *Ieee Access* **6**, 665–676 (2017).

[13] Wei, Y. & Wu, D. Conditional variational transformer for bearing remaining useful life prediction. *Advanced Engineering Informatics* **59**, 102247 (2024).

[14] Neupane, D. & Seok, J. Bearing fault detection and diagnosis using case western reserve university dataset with deep learning approaches: A review. *Ieee Access* **8**, 93155–93178 (2020).

[15] Hendriks, J., Dumond, P. & Knox, D. Towards better benchmarking using the cwru bearing fault dataset. *Mechanical Systems and Signal Processing* **169**, 108732 (2022).

[16] Song, X., Liao, Z., Jia, B., Kong, D. & Niu, J. Rolling bearing fault diagnosis under different severity based on statistics detection index and canonical discriminant analysis. *IEEE Access* (2023).

[17] Hong, H. & Liang, M. Fault severity assessment for rolling element bearings using the lempel-ziv complexity and continuous wavelet transform. *Journal of sound and vibration* **320**, 452–468 (2009).

[18] Song, W., Liu, H. & Zio, E. Long-range dependence and heavy tail characteristics for remaining useful life prediction in rolling bearing degradation. *Applied Mathematical Modelling* **102**, 268–284 (2022).

[19] Wei, Y. & Wu, D. Remaining useful life prediction of bearings with attention-awared graph convolutional network. *Advanced Engineering Informatics* **58**, 102143 (2023).

[20] Wang, J., Liang, Y., Zheng, Y., Gao, R. X. & Zhang, F. An integrated fault diagnosis and prognosis approach for predictive maintenance of wind turbine bearing with limited samples. *Renewable energy* **145**, 642–650 (2020).

[21] Hu, C. et al. *Engineering design under uncertainty and health prognostics* (Springer, 2019).

[22] Bond, L. J. From ndt to prognostics: advanced technologies for improved quality, safety and reliability. In *2015 IEEE Far East NDT New Technology & Application Forum (FENDT)*, 1–9 (IEEE, 2015).

[23] Hamadache, M., Jung, J. H., Park, J. & Youn, B. D. A comprehensive review of artificial intelligence-based approaches for rolling element bearing phm: Shallow and deep learning. *JMST Advances* **1**, 125–151 (2019).

[24] Xu, D. & Li, C. Optimization of deep belief network based on sparrow search algorithm for rolling bearing fault diagnosis. *IEEE Access* (2024).

[25] Lei, Y. et al. Applications of machine learning to machine fault diagnosis: A review and roadmap. *Mechanical Systems and Signal Processing* **138**, 106587 (2020).

[26] Ruan, D., Wang, J., Yan, J. & Gühmann, C. Cnn parameter design based on fault signal analysis and its application in bearing fault diagnosis. *Advanced Engineering Informatics* **55**, 101877 (2023).

[27] Case western reserve university bearing data center website. accessed: Aug. 06, 2023. [online]. available at: https://engineering.case.edu/bearingdatacenter/download-data-file.

[28] Lessmeier, C., Kimotho, J. K., Zimmer, D. & Sextro, W. Condition monitoring of bearing damage in electromechanical drive systems by using motor current signals of electric motors: A benchmark data set for data-driven classification. **3** (2016).

[29] O’Mahony, N. et al. Deep learning vs. traditional computer vision. In *Advances in Computer Vision: Proceedings of the 2019 Computer Vision Conference (CVC), Volume 1 1*, 128–144 (Springer, 2020).

[30] Khan, S. A. & Kim, J.-M. Automated bearing fault diagnosis using 2d analysis of vibration acceleration signals under variable speed conditions. *Shock and Vibration* **2016**, 8729572 (2016).

[31] Khan, S. A. & Kim, J.-M. Rotational speed invariant fault diagnosis in bearings using vibration signal imaging and local binary patterns. *The Journal of the Acoustical Society of America* **139**, EL100–EL104 (2016). 10.1121/1.4945818 27106344

[32] Kaplan, K., Kaya, Y., Kuncan, M., Minaz, M. R. & Ertunç, H. M. An improved feature extraction method using texture analysis with lbp for bearing fault diagnosis. *Applied Soft Computing* **87**, 106019 (2020).

[33] Kuncan, M. An intelligent approach for bearing fault diagnosis: combination of 1d-lbp and gra. *Ieee Access* **8**, 137517–137529 (2020).

[34] Wu, W. Fault monitoring and diagnosis of motor operation status based on lbp-svm. *IEEE Access* (2024).

[35] Wang, S. et al. Matching demodulation transform and synchrosqueezing in time-frequency analysis. *IEEE Transactions on Signal Processing* **62**, 69–84 (2013).

[36] Bessous, N., Zouzou, S., Bentrah, W., Sbaa, S. & Sahraoui, M. Diagnosis of bearing defects in induction motors using discrete wavelet transform. *International Journal of System Assurance Engineering and Management* **9**, 335–343 (2018).

[37] Bentrah, W., Bessous, N., Sbaa, S., Pusca, R. & Romary, R. A comparative study between the adaptive wavelet transform and dwt methods applied to a outer raceway fault detection in induction motors based on the frequencies analysis. In *2020 International Conference on Electrical Engineering (ICEE)*, 1–7 (IEEE, 2020).

[38] Chennana, A. *et al.* A comparative study between emd-mckd and emd-meda techniques for the bearing faults diagnosis in induction motors. In *2023 International Conference on Electrical Engineering and Advanced Technology (ICEEAT)*, vol. 1, 1–6 (IEEE, 2023).

[39] Ahmia, A., Megherbi, A. C., Sbaa, S., Bessous, N. & Chennana, A. Improvement of the mckd process using emd and feature mode decomposition under bearing damage in induction motors: Comparison study. In *2023 2nd International Conference on Electronics, Energy and Measurement (IC2EM)*, vol. 1, 1–5 (IEEE, 2023).

[40] Damine, Y. et al. A new bearing fault detection strategy based on combined modes ensemble empirical mode decomposition, kmad, and an enhanced deconvolution process. *Energies* **16**, 2604 (2023).

[41] Chennana, A. et al. A bearing faults diagnosis enhancement using emd and meda. In *2024 2nd International Conference on Electrical Engineering and Automatic Control (ICEEAC)*, 1–6 (IEEE, 2024).

[42] Wang, N. *International Journal of Computer Science and Information Technology* **2**, 100–109 (2024).

[43] Chang, Y. & Bao, G. Enhancing rolling bearing fault diagnosis in motors using the ocssa-vmd-cnn-bilstm model: A novel approach for fast and accurate identification. *IEEE Access* (2024).

[44] Nishat, Toma R. & Kim, J.-M. Bearing fault classification of induction motors using discrete wavelet transform and ensemble machine learning algorithms. *Applied Sciences* **10**, 5251 (2020).

[45] Siddique, M. F. et al. Advanced bearing-fault diagnosis and classification using mel-scalograms and fox-optimized ann. *Sensors* **24**, 7303 (2024). 39599080 10.3390/s24227303 PMC11598819

[46] Ullah, N., Umar, M., Kim, J.-Y. & Kim, J.-M. Enhanced fault diagnosis in milling machines using cwt image augmentation and ant colony optimized alexnet. *Sensors (Basel, Switzerland)* **24**, 7466 (2024). 39686003 10.3390/s24237466 PMC11644436

[47] Avendano, D. N., Deschrijver, D. & Van Hoecke, S. Transfer learning for anomaly detection using bearings’ vibration signals. *International Journal of Acoustics and Vibration* **28**, 420–434 (2023).

[48] Boashash, B. *Time-frequency signal analysis and processing: a comprehensive reference* (Academic press, 2015).

[49] Mohd Ghazali, M. H. & Rahiman, W. Vibration analysis for machine monitoring and diagnosis: a systematic review. *Shock and Vibration* **2021**, 9469318 (2021).

[50] Lu, D. & Weng, Q. A survey of image classification methods and techniques for improving classification performance. *International journal of Remote sensing* **28**, 823–870 (2007).

[51] Logan, B. et al. Mel frequency cepstral coefficients for music modeling. In *Ismir*, vol. 270, 11 (Plymouth, MA, 2000).

[52] O’Shaughnessy, D. Linear predictive coding. *IEEE potentials* **7**, 29–32 (1988).

[53] Todisco, M., Delgado, H. & Evans, N. Constant q cepstral coefficients: A spoofing countermeasure for automatic speaker verification. *Computer Speech & Language* **45**, 516–535 (2017).

[54] Ahonen, T., Hadid, A. & Pietikainen, M. Face description with local binary patterns: Application to face recognition. *IEEE transactions on pattern analysis and machine intelligence* **28**, 2037–2041 (2006). 17108377 10.1109/TPAMI.2006.244

[55] Zhang, B., Gao, Y., Zhao, S. & Liu, J. Local derivative pattern versus local binary pattern: face recognition with high-order local pattern descriptor. *IEEE transactions on image processing* **19**, 533–544 (2009). 19887313 10.1109/TIP.2009.2035882

[56] Ojansivu, V. & Heikkilä, J. Blur insensitive texture classification using local phase quantization. In *Image and Signal Processing: 3rd International Conference, ICISP 2008. Cherbourg-Octeville, France, July 1-3, 2008. Proceedings 3*, 236–243 (Springer, 2008).

[57] Hershey, S. et al. Cnn architectures for large-scale audio classification. In *2017 ieee international conference on acoustics, speech and signal processing (icassp)*, 131–135 (IEEE, 2017).

[58] Di Maggio, L. G. Intelligent fault diagnosis of industrial bearings using transfer learning and cnns pre-trained for audio classification. *Sensors* **23**, 211 (2022). 36616809 10.3390/s23010211 PMC9823443

[59] Simonyan, K. Very deep convolutional networks for large-scale image recognition. arXiv preprint arXiv:1409.1556 (2014).

[60] Tsalera, E., Papadakis, A. & Samarakou, M. Comparison of pre-trained cnns for audio classification using transfer learning. *Journal of Sensor and Actuator Networks* **10**, 72 (2021).

[61] Howard, A. G. et al. Mobilenets: Efficient convolutional neural networks for mobile vision applications. arXiv preprint arXiv:1704.04861 (2017).

[62] Gemmeke, J. F. et al. Audio set: An ontology and human-labeled dataset for audio events. In *2017 IEEE international conference on acoustics, speech and signal processing (ICASSP)*, 776–780 (IEEE, 2017).

[63] Chen, W., Kamachi, H., Yokokubo, A. & Lopez, G. Bone conduction eating activity detection based on yamnet transfer learning and lstm networks. In *BIOSIGNALS*, 74–84 (2022).

[64] Zhang, T., Fang, B., Tang, Y. Y., Shang, Z. & Xu, B. Generalized discriminant analysis: A matrix exponential approach. *IEEE Transactions on Systems, Man, and Cybernetics, Part B (Cybernetics)* **40**, 186–197 (2009). 10.1109/TSMCB.2009.2024759 19651556

[65] Xanthopoulos, P. et al. Linear discriminant analysis. *Robust data mining* 27–33 (2013).

[66] Adil, M., Abid, M., Khan, A. Q., Mustafa, G. & Ahmed, N. Exponential discriminant analysis for fault diagnosis. *Neurocomputing* **171**, 1344–1353 (2016).

[67] Ouamane, A., Messaoud, B., Guessoum, A., Hadid, A. & Cheriet, M. Multi scale multi descriptor local binary features and exponential discriminant analysis for robust face authentication. In *2014 IEEE International conference on image processing (ICIP)*, 313–317 (IEEE, 2014).

[68] Kuang, H., Hang Chan, L. L., Liu, C. & Yan, H. Fruit classification based on weighted score-level feature fusion. *Journal of Electronic Imaging* **25**, 013009–013009 (2016).

[69] Dong, Y., Liu, Q., Du, B. & Zhang, L. Weighted feature fusion of convolutional neural network and graph attention network for hyperspectral image classification. *IEEE Transactions on Image Processing* **31**, 1559–1572 (2022). 35077363 10.1109/TIP.2022.3144017

[70] Rezazadeh, N., Perfetto, D., de Oliveira, M., De Luca, A. & Lamanna, G. A fine-tuning deep learning framework to palliate data distribution shift effects in rotary machine fault detection. *Structural Health Monitoring* 14759217241295951 (2024).

[71] Niyongabo, J., Zhang, Y. & Ndikumagenge, J. Bearing fault detection and diagnosis based on densely connected convolutional networks. *acta mechanica et automatica* **16**, 130–135 (2022).

[72] Makrouf, I., Zegrari, M., Ouachtouk, I. & Dahi, K. Multi-source information fusion fault diagnosis for rotating machinery using signal and data processing. In *Surveillance, Vibrations, Shock and Noise* (2023).

[73] Li, Z. et al. Tdanet: A novel temporal denoise convolutional neural network with attention for fault diagnosis. arXiv preprint arXiv:2403.19943 (2024).

[74] Su, K., Liu, J. & Xiong, H. Hierarchical diagnosis of bearing faults using branch convolutional neural network considering noise interference and variable working conditions. *Knowledge-Based Systems* **230**, 107386 (2021).

[75] Gao, T., Yang, J. & Tang, Q. A multi-source domain information fusion network for rotating machinery fault diagnosis under variable operating conditions. *Information Fusion* **106**, 102278 (2024).

[76] Li, Q. et al. Fault diagnosis of nuclear power plant sliding bearing-rotor systems using deep convolutional generative adversarial networks. *Nuclear Engineering and Technology* (2024).

[77] Wang, H. & Zhang, X. Fault diagnosis using imbalanced data of rolling bearings based on a deep migration model. *IEEE Access* (2024).

[78] Lee, C.-Y. & Maceren, E. D. C. Induction motor bearing fault classification using deep neural network with particle swarm optimization-extreme gradient boosting. *IET Electric Power Applications* **18**, 297–311 (2024).

[79] Gao, H., Ma, J., Zhang, Z. & Cai, C. Bearing fault diagnosis method based on attention mechanism and multi-channel feature fusion. *IEEE Access* (2024).

